# Integrated Analysis of lncRNAs, mRNAs, and TFs to Identify Regulatory Networks Underlying MAP Infection in Cattle

**DOI:** 10.3389/fgene.2021.668448

**Published:** 2021-07-05

**Authors:** Maryam Heidari, Abbas Pakdel, Mohammad Reza Bakhtiarizadeh, Fariba Dehghanian

**Affiliations:** ^1^Department of Animal Sciences, College of Agriculture, Isfahan University of Technology, Isfahan, Iran; ^2^Department of Animal and Poultry Science, College of Aburaihan, University of Tehran, Tehran, Iran; ^3^Department of Biology, University of Isfahan, Isfahan, Iran

**Keywords:** MAP infection, Johne’s disease, weighted gene co-expression network, lncRNA-mRNA-TF networks, RNA-seq, hub genes, hub-hub genes

## Abstract

Johne’s disease is a chronic infection of ruminants that burdens dairy herds with a significant economic loss. The pathogenesis of the disease has not been revealed clearly due to its complex nature. In order to achieve deeper biological insights into molecular mechanisms involved in MAP infection resulting in Johne’s disease, a system biology approach was used. As far as is known, this is the first study that considers lncRNAs, TFs, and mRNAs, simultaneously, to construct an integrated gene regulatory network involved in MAP infection. Weighted gene coexpression network analysis (WGCNA) and functional enrichment analysis were conducted to explore coexpression modules from which nonpreserved modules had altered connectivity patterns. After identification of hub and hub-hub genes as well as TFs and lncRNAs in the nonpreserved modules, integrated networks of lncRNA-mRNA-TF were constructed, and cis and trans targets of lncRNAs were identified. Both cis and trans targets of lncRNAs were found in eight nonpreserved modules. Twenty-one of 47 nonpreserved modules showed significant biological processes related to the immune system and MAP infection. Some of the MAP infection’s related pathways in the most important nonpreserved modules comprise “positive regulation of cytokine-mediated signaling pathway,” “negative regulation of leukocyte migration,” “T-cell differentiation,” “neutrophil activation,” and “defense response.” Furthermore, several genes were identified in these modules, including SLC11A1, MAPK8IP1, HMGCR, IFNGR1, CMPK2, CORO1A, IRF1, LDLR, BOLA-DMB, and BOLA-DMA, which are potentially associated with MAP pathogenesis. This study not only enhanced our knowledge of molecular mechanisms behind MAP infection but also highlighted several promising hub and hub-hub genes involved in macrophage-pathogen interaction.

## Introduction

Johne’s disease (JD) is a chronic granulomatous intestinal infection in ruminant animals caused by *Mycobacterium avium* subsp. *paratuberculosis* (MAP). Clinical signs of JD include diarrhea, weight loss, decreased milk production, and mortality (Li et al., 2005). According to a research in the United States, 91% of dairy herds are infected with MAP (Lombard et al., 2013), but the point is that most of the animals in a herd usually are in a silent phase of the disease and do not show any symptoms until they reach a clinical stage. The infection will be detectable only in the clinical stage, which will happen between the ages of 2–5 years in dairy cattle. This long latent phase is considered a basic problem of the disease checking and controlling (Marino et al., 2017).

JD is a multifactorial infection, and its etiology and the underlying molecular mechanisms have not been completely elucidated. Hence, identification of key genes and pathways involved in this infection can be a helpful approach to prevent the clinical events. In this context, most previous studies focused on the screening of the differentially expressed genes. Machugh et al. (2012) investigated pan-genomic gene expression in bovine monocyte-derived macrophages in response to MAP infection in different times after infection. They identified the genes involved in the inflammatory response, cellular signaling pathways, and apoptosis (Machugh et al., 2012). David et al. (2014) reported downregulation of BNBD9-Like, s100A9, GPR77, and C5a2 genes at 6 months after MAP infection and downregulation of BOLA/MHC-1 at 9 months after infection (David et al., 2014). Thirunavukkarasu et al. (2014) identified host genes involved in cholesterol homeostasis, calcium homeostasis, and antibacterial defense mechanisms, which were downregulated in response to MAP exposure (Thirunavukkarasu et al., 2014). Shin et al. (2015) determined the major gene networks and important pathways caused the immune response to MAP infection. The results of Shin et al. (2015) study indicated downregulation of production and metabolism of reactive oxygen species, activation of pathways related to the host-defense response against MAP and anti-inflammatory response in different groups of infected animals (Shin et al., 2015). The results of Hempel et al. (2016) research showed that significant differentially expressed genes between infected and control cows were enriched in many pathways associated with the immune system such as T- and B-cell receptor signaling, apoptosis, NOD-like receptor signaling, and leukocyte migration pathways (Hempel et al., 2016). Park et al. (2016) suggested six genes including LTF, HGF, HP, CXCR3, GBP6, and TFRC that play major roles in the host immune response to MAP during the subclinical stage (Park et al., 2016).

Despite these studies, it is well known that considering the modules of genes than individual assessment of the genes may better explain the complex etiology of diseases (Bakhtiarizadeh et al., 2018; Bakhtiarizadeh et al., 2020). On the other hand, integrated regulatory networks that describe the relationships among different types of regulators (such as noncoding RNAs and transcription factors (TFs)), simultaneously, can provide a broader and deeper insights to understand the molecular mechanisms involved in the disease or traits of interest. In this regard, long noncoding RNAs (lncRNAs) are one of the main classes of noncoding RNAs with more than 200 nucleotides and can regulate a variety of physiological functions in connection with other molecules. It is reported that lncRNAs have a vital role in the regulation of eukaryotic gene expression. The specific role of lncRNAs in the host cellular response to bacterial infections is pointed out in several researches (Zur Bruegge et al., 2017). Furthermore, it is demonstrated that lncRNAs are associated with the fine-tuned regulation of inflammatory processes (Mathy and Chen, 2017; Chew et al., 2018). Moreover, TFs are essential regulators of gene expression in a cell and control gene activity through binding to the promoter regions of their target genes (Bakhtiarizadeh et al., 2014). Based on the results of the study of Gupta et al. (2019) on MAP infection, coexpression analysis of lncRNAs and their neighboring coding genes propose regulatory functions of lncRNAs in the pathways related to immune response (Gupta et al., 2019). Ibeagha-Awemu et al. (2018) used weighted gene coexpression network analysis (WGCNA) to identify genes and pathways regulating MAP infection. According to their results, two coexpressed modules were related to JD, and CTSH and MERTK hub genes were involved in degradation of lysosomal proteins and phagocytosis of apoptotic cells, respectively (Ibeagha-Awemu et al., 2018). Malvisi et al. (2016) investigated mRNA and miRNA expression in MAP-positive and MAP-negative Holstein cows. Their results of miRNA level analysis was indicative of the correlation of target genes involved in immune process and the role of miRNAs in regulation of host response to MAP infection (Malvisi et al., 2016).

To the best of our knowledge, there is no study that considers lncRNAs, TFs, and mRNAs, simultaneously, to construct an integrated gene regulatory network involved in MAP infection. Furthermore, the role of lncRNAs in the pathogenesis of JD remains largely unclear. Therefore, construction of such a network can improve our understanding of the pathogenesis of JD. In this respect, WGCNA is the most widely used approach in systems biology that can construct gene coexpression networks. WGCNA is an advanced data mining approach to find the modules of highly correlated gene and related hub genes within each module. The main assumption behind this method is a module of highly connected genes which are coordinated in terms of expression that are probably functionally related to each other (Bakhtiarizadeh et al., 2018). Hence, this approach enables us to cluster the genes into modules and associate the modules to biological functions and regulatory mechanisms. In the present study, a system of biology approach was applied using WGCNA method and RNA sequencing data to construct an integrated gene regulatory network involved in MAP infection in monocyte-derived macrophages. This approach enabled us to (1) construct a coexpression network and finding important modules, (2) explore a lncRNA-TF-mRNA network, (3) identify hub genes within each module that may be associated with the infection, and (4) annotate the modules by functional enrichment analysis.

## Materials and Methods

### Dataset

RNA-Seq data (accession number GSE62048) was downloaded from the gene expression omnibus (GEO) database of the National Center for Biotechnology Research (NCBI). The data contained 35 monocyte-derived macrophage samples obtained from seven Holstein-Friesian cows and were infected *in vitro* with a clinical isolate of MAP. The animals did not have a recent history of JD and were also negative for infection with *Mycobacterium bovis* (Casey et al., 2015). The samples were related to two groups including 21 controls (at 0, 2, and 6 h after infection with MAP) and 14 infected (at 2 and 6 h after infection with MAP) samples. All 35 RNA samples were single end and strand specific, which were sequenced using Illumina HiSeq 2000.

### RNA-Seq Data Analysis

FastQC software v0.11.5 was used to check the quality of the raw reads (Andrews, 2010). Then, low-quality reads were filtered or trimmed with Trimmomatic software v0.38 (Bolger et al., 2014). Hisat2 software v2.0.4 (Kim et al., 2019) was applied to align clean reads to the bovine reference genome (ARS-UCD1.2 from Ensemble database). Finally, Htseq-count software v0.6.1 was used to determine counts of reads mapped to annotated genes based on the ENSEBMBL bovine GTF file (version 98) (Anders et al., 2015).

### Coexpression Gene Network Construction

The expression matrix created in the previous step was used for WGCNA according to the standard WGCNA R package procedure (Langfelder and Horvath, 2008). Before starting WGCNA, the raw count matrix was normalized using voom function of limma package of R software. Only the genes that had expressions of greater or equal to 1 count per million reads (CPM) in at least five samples were kept for further analysis. Also, the genes with standard deviations greater than 0.25 across the samples were considered for the next stages of analysis. Finally, the genes were scaled so that they had average = 0 and standard deviation = 1.

Since coexpression analysis is very sensitive to outliers, the expression matrix should be first checked if there are any outlying samples. The adjacency function in the WGCNA package was used to calculate distance-based adjacency matrices to identify possible outlying samples, and the samples with a standardized connectivity score of less than −2.5 were removed. The adjacency matrix is constructed based on the correlations among the gene expression profiles (Horvath, 2011). Bi-weight mid-correlation was used to obtain pairwise correlations among the genes since it combines advantages of the Pearson correlation (relatively high power) and the Spearman correlation (relatively high robustness) (Hardin et al., 2007; Wilcox, 2011). The final dataset was used in WGCNA to build a signed weighted network. In fact, a signed network considers the sign of the underlying correlation coefficient. In addition, it has been demonstrated that by using signed networks, it is possible to identify modules with more significant enrichment of functional groups (Langfelder and Horvath, 2008; Smita et al., 2013). Then goodSamplesGenes function of the WGCNA package was applied to remove genes and samples with too many missing values. Based on pickSoftThreshold function of WGCNA package, a soft threshold power was set at *β* = 19 correspondent to a scale-free topology index (*R*^2^) of ≥ 0.8 (Supplementary File 1), to ensure a scale-free network. Scale free is an important property of the biological gene networks and indicates that some genes are more connected than others (Horvath, 2011).

### Module Detection

Based on the power of 19, the adjacency matrix was replaced with a weighted adjacency matrix and transformed into a topological overlap matrix (TOM). The TOM measures interconnectedness property of the network (Horvath, 2011). Hierarchical clustering was applied to classify genes with similar expression profiles into the same modules, based on the TOM dissimilarity. Then the coexpressed modules were identified through a dynamic hybrid tree cut algorithm. Finally, the modules with high correlated eigengenes were merged so that the merge cut height was 0.25 (Horvath, 2011). BlockwiseModules function of the WGCNA package was used to detect modules. The gene expression profiles of each module were summarized by module eigengenes, and the similarities among the modules were estimated by this parameter. Module eigengene is defined as the first principal component of a given module (Langfelder and Horvath, 2008). Module eigengenes for all modules were calculated by the moduleEigengenes function of the WGCNA package.

### Preservation Analysis

Module preservation analysis was performed by considering normal samples as reference set and Zsummary and medianRank criteria. Higher Zsummary implies strong evidence for module preservation. However, since Zsummary often depends on the module size, and it was required to compare modules with different sizes, medianRank which is not affected by module size was also used. Unlike Zsummary, a module with lower medianRank displays stronger preservation statistics than a module with a higher medianRank. In the present study, modules with Zsummary < 10 or medianRank ≥ 8 were considered nonpreserved modules (Horvath, 2011).

### Construction of lncRNA-mRNA-TF Networks and Finding Hub and Hub-Hub Genes

A protein-protein interaction (PPI) network provides details on the functional interactions among proteins. The construction of the PPI network was done using STRING database (Szklarczyk et al., 2019). Since lncRNAs were not considered in the STRING database, lncRNA interactions, which were calculated using the WGCNA R package, were added to establish an integrated network. TF genes were also identified using the AnimalTFDB webpage (Zhang et al., 2012). Finally, lncRNA-mRNA-TF networks were visualized using Cytoscape software (Shannon et al., 2003).

It is well known that, in a given module, the highly connected genes (hub genes) have stronger correlations with certain biological functions. These genes tend to have high module membership values to the respective module. Intramodular connectivity (K_ME_ orK_IM_) were used as quantitative measures of module membership (Horvath, 2011). K_ME_ was used to identify hub genes and genes with |K_ME_| ≥ 0.7 were considered the hub genes of the respective module. It is demonstrated that intramodular hub genes are highly correlated with the module eigengene, which is referred to a representative of the gene expression profiles in a module (Langfelder and Horvath, 2008). The identified hub genes in each module were subjected to PPI network construction using the STRING database. Among the hub genes, the genes with the highest connections were defined as hub-hub genes. In order to obtain hub-hubs, after calculation of PPIs of hub genes in each module, cytoHubba plugin of Cytoscape software was applied. This plugin ranks the nodes, i.e., hub genes, in a network by their network features. CytoHubba provides 12 topological analysis methods including Maximal Clique Centrality (MCC), Density of Maximum Neighborhood Component Degree (DMNC), Maximum Neighborhood Component (MNC), Degree, Edge Percolated Component (EPC), Bottleneck, EcCentricity, Closeness, Radiality, Betweenness, Stress, and Clustering Coefficient to explore important nodes in the biological networks (Chin et al., 2014). Here, our previous approach was applied to find hub-hubs (Bakhtiarizadeh et al., 2020) and results of 12 methods were aggregated using the RankAggreg package of R software. The RankAggreg package combines the created ranks using two methods consisting of the Cross-Entropy Monte Carlo algorithm and the Genetic Algorithm (Pihur et al., 2009), which leads to hub-hub genes. Totally, about 25% of the hub genes of each module were accounted as hub-hubs. Finally as explained earlier, the interactions of the hub lncRNAs with other hub genes were added to STRING results. Furthermore, the potential target genes of lncRNAs were identified by searching the protein-coding genes located in 100 kb upstream or downstream of each lncRNA and the nearest gene was considered cis target. On the other hand, all the mRNA genes in a given module were considered trans target genes of the lncRNAs in that module.

### Functional Enrichment Analysis

Functional enrichment analysis was performed using ViSEAGO package of R software. ViSEAGO is a data mining of biological functions to facilitate functional gene ontology (GO) analysis of complex experimental design with multiple comparisons of interest. ViSEAGO extends classical functional GO analysis to focus on functional coherence by aggregating closely related biological themes while studying multiple datasets at once (Brionne et al., 2019). Through this analysis, the possible functions of the genes in a given module were predicted. *p*-value ≤0.01 was chosen to identify significant outcome. Biological process was used to interpret the GO results.

## Results

### Summary of the RNA-Seq Data

**FIGURE 1:**
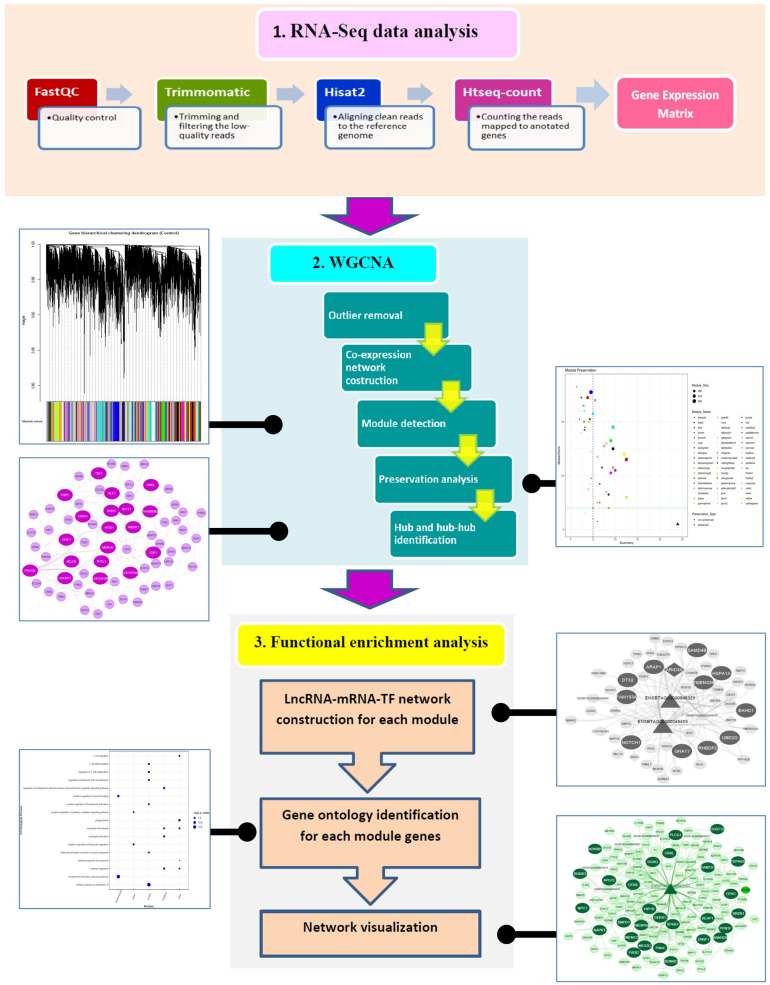
The pipeline of the methodology used in the present study.

The workflow of the present study is illustrated in Figure 1. Overall, 693,703,609 raw reads from 35 RNA-Seq samples were analyzed, and 634,958,235 clean reads were the results of trimming. On average, 76% of the clean reads were mapped to reference genome. The summary of the RNA-Seq analysis results and the alignment of all samples to reference genome are provided in Supplementary File 2. A total number of 11,373 genes were identified to have expression equal to/greater than 1 CPM in at least five samples. After removing the genes with low variance, 11,013 genes were retained for further analysis. Of these, 10,806 and 109 genes were mRNAs and lncRNAs, respectively. Out of mRNA genes, 634 genes were detected to be TFs. The gene expression matrix of the samples in normal and MAP-infected groups is available in Supplementary File 3.

### Module Detection

**FIGURE 2:**
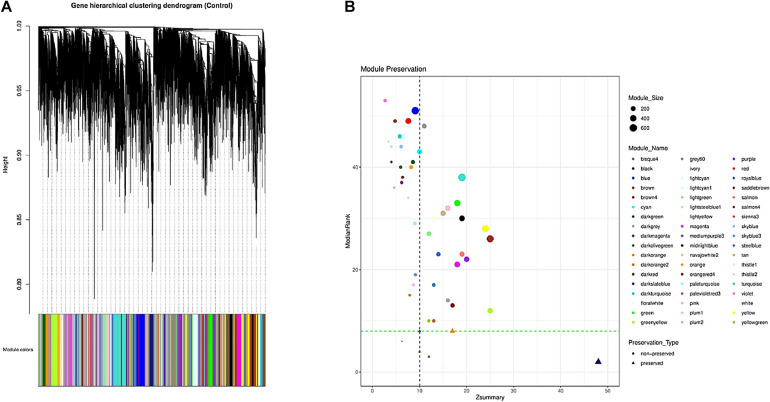
Identification of the gene coexpression modules using hierarchical clustering; the *x*-axis and *y*-axis indicate the genes and coexpression distance, respectively, and each color represents a module **(A)**. Preservation analysis based on the MedianRank and Zsummary criteria **(B)**.

Overall 56 coexpression modules were identified in normal samples through hierarchical clustering based on the topological overlap matrix (TOM) dissimilarity measure (Figure 2A). Of these, gray module (related to background genes) and gold module (including a random sample of genes) were removed. In the remaining 54 modules, the number of genes per module ranged from 35 in palevioletred3 and navajowhite2 modules to 710 in the turquoise module. The average module size was about 190 genes (Supplementary File 4).

### Hub Genes Identification

The complete list of the hub genes in each module is presented in Supplementary File 5. The highest and the lowest numbers of hub genes were found in the yellow module (211) and palevioletred3 module (18), respectively.

### Preservation Analysis

To investigate if the modular structure of the normal samples were preserved in the infected samples, the preservation status of the modules was checked (Figure 2B). Preservation analysis indicated that seven modules including floralwhite, midnightblue, salmon4, bisque4, thistle1, darkorange, and darkslateblue with Zsummary > 10 and medianRank ≤ 8 were preserved, while 47 modules were nonpreserved (Supplementary File 6). Since connectivity patterns in the nonpreserved modules are different in normal compared with infected samples, they can indicate sets of genes influenced by disease.

### Enrichment Analysis

#### Preserved Modules

**FIGURE 3:**
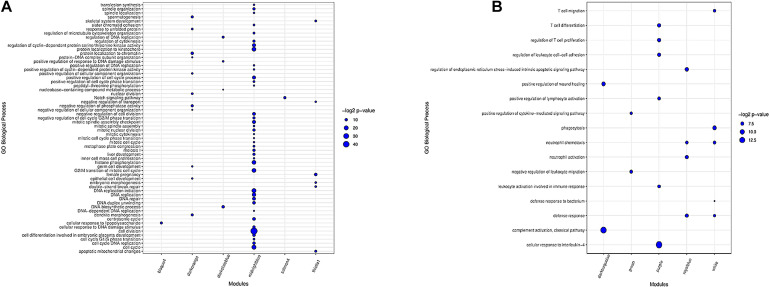
Biological processes of GO analysis for genes in the preserved modules **(A)**. Biological processes of GO analysis for genes in the most important nonpreserved modules **(B)**. The size of the points represents –log2 *p*-value of genes associated with each term.

In total, 16 clusters of gene ontology (GO) terms related to 62 biological processes were identified in seven preserved modules (Supplementary File 7). The most significantly enriched GO terms belonged to midnightblue (39 terms) and darkorange (11 terms) modules. Genes in thistle1 and darkslateblue modules were significantly enriched in six and four biological processes, respectively. There was only one significant GO term in salmon4 module and bisque4 module. Floralwhite module did not show any significant GO terms. The results of the enrichment analysis in the preserved modules are presented in Figure 3A. The complete list of the functional enrichment analysis for preserved modules is available in Supplementary File 8.

#### Nonpreserved Modules

Functional enrichment analysis revealed 42 clusters of gene ontology terms related to 312 biological processes in the non-preserved modules that are provided in Supplementary File 9. The most significantly enriched GO terms were found in grey60 module (24 terms), and several modules including salmon, yellow, darkred, turquoise, white, purple, black, greenyellow, royalblue, lightgreen, and sienna3 had more than 12 significant GO terms. Lightsteelblue1 and violet modules did not show any significant terms. The results of enrichment analysis in the nonpreserved modules of interest are represented in Figure 3B and Supplementary File 10. The complete list of the functional enrichment analysis for nonpreserved modules is available in Supplementary File 11.

### LncRNAs and TFs

Out of 47 nonpreserved modules, 32 and 43 modules contained lncRNAs and TFs, respectively. The range of lncRNA number was between one and eight, and the number of TFs changed from one to 77 genes in different nonpreserved modules. The blue module and the turquoise module had the highest number of lncRNAs and TFs, respectively. The complete list of TFs and lncRNAs of all modules are available in Supplementary Files 12, 13, respectively. In all 32 modules containing lncRNA, cis targets in upstream and downstream of their respective lncRNAs were identified (Supplementary File 14), while in some of these nonpreserved modules including brown, darkgray, green, lightyellow, red, royalblue, salmon, and turquoise, lncRNAs had trans targets too (Supplementary File 15). We hypothesized that the predicted cis and trans target genes can be potentially regulated by the respective lncRNAs.

### Integrated Networks and Hub-Hub Genes

**FIGURE 4:**
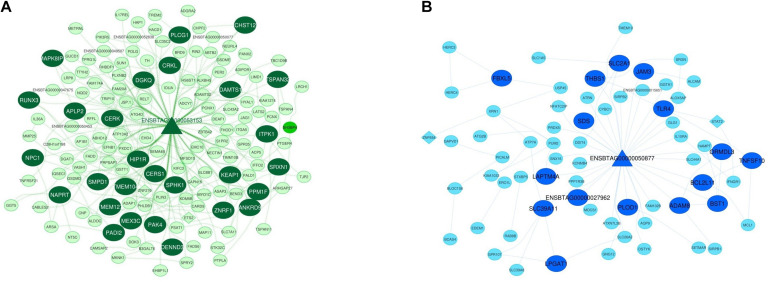
Integrated regulatory network of green module. The dark green color represents hub-hub genes, and the light green color represents hub genes **(A)**. Integrated regulatory network of royal blue module. The royal blue color represents hub-hub genes, and the blue color represents hub genes **(B)**. In both networks, each node represents a gene and each edge represents the interaction between genes. mRNAs, lncRNAs, and TFs are indicated with circles, triangles, and diamond shapes, respectively.

Considering the worth of the hub and hub-hub genes, a PPI network was constructed to evaluate the interactions between these genes in each nonpreserved module. The mRNA-lncRNA-TF networks of the most important nonpreserved modules including green module and royalblue module are shown in Figures 4A,B, respectively. The mRNA-lncRNA-TF networks of the nonpreserved modules of interest are presented in Supplementary File 16. In addition, the complete list of the hub-hub genes in the nonpreserved modules is presented in Supplementary File 17.

## Discussion

JD is a chronic disease caused by MAP with a complex and relatively unknown nature, which adversely affects performance traits and causes health problems. In the present study, integrated regulatory networks were constructed using the WGCNA approach to achieve a more comprehensive knowledge of molecular mechanisms involved in MAP infection. This approach allowed us to identify some modules of coexpressed genes related to immune response as some of them were already reported as important genes in MAP infection in macrophages, which confirms the reliability of our approach. Furthermore, identification of hub and hub-hub genes enriched for the biological processes related to the response of the macrophages to MAP infection make them attractive candidates to be used as diagnostic biomarkers of MAP infection. Hubs, especially hub-hubs, are frequently more relevant to the network’s functionality than other nodes (Albert et al., 2000). In fact, these genes have the highest intra-modular connectivity and can be potentially considered as disease-associated markers (Horvath, 2011). In this regard, hub and hub-hub genes were focused more than others.

The present study on the strength of WGCNA fully took advantage of the whole transcriptome and produced more complete results than studies that only consider differentially expressed genes. In a module, highly coexpressed genes are more likely to be functionally associated (Bakhtiarizadeh et al., 2018). In the preserved modules, as expected, the enriched terms were frequently related to the general processes of cells that usually occur in all cells. So, the genes in the preserved modules were not affected by MAP infection and mostly regulate the main cellular steps such as cell division, cell cycle, nuclear division, DNA biosynthetic process, DNA replication, and histone phosphorylation. On the other hand, considering the fact that MAP infection affects the immune system, the genes involved in the immune systems in infected animals are more influenced. In this context, module preservation analysis supply information about whether the properties of a module in a network are altered under different conditions (normal vs. MAP infected). Hence, it is expected to observe the nonpreserved modules that are enriched for the genes related to these biological processes, which indicate sets of genes influenced by MAP infection. Out of 42 created clusters (clusters of biological processes) in the nonpreserved modules, 13 clusters (including 26 biological processes) were related to the immune system. Totally, 21 nonpreserved modules had significant biological processes associated with immune system (*p*-value <0.01). Interestingly, some of the genes in the nonpreserved modules were reported to be associated with MAP infection in the previous studies that investigated the response of the macrophages to an in vitro MAP infection (Ruiz-Larrañaga et al., 2010; Marino et al., 2017; Ariel et al., 2019; Johansen et al., 2019). Detailed explanations of the 21 nonpreserved modules are provided in Supplementary File 18. Here, five nonpreserved modules that were considered most important ones based on their biological processes related to MAP infection, and relevant genes were discussed in detail. Further information regarding these five modules is also available in Supplementary File 18.

### Green Module

Functional enrichment analysis suggested that the green module was enriched in “positive regulation of cytokine-mediated signaling pathway” and “negative regulation of leukocyte migration.” When MAP enters the animal’s cell, it is phagocytized by macrophages on the submucosal part of the intestinal epithelium. MAP can survive and proliferate within phagosomes and prevents apoptosis and phagosomal maturation in infected macrophages (Marino et al., 2017). In this step, macrophages should be activated to increase their ability for killing intracellular MAP and controlling the infection. The activation will be achieved *via* the production of gamma interferon (IFN-γ) and other cytokines by Th1-type T-helper lymphocytes (Zurbrick et al., 1988; Khalifeh and Stabel, 2004). In fact, MAP-infected macrophages secrete proinflammatory cytokines, which activate an early protective Th1 response. The proinflammatory cytokines expression in the MAP-infected host is critical in both protective immunity and MAP survival (Casey et al., 2015). Hence, the green module represented the functional enriched terms participating in MAP infection related pathways. The role of many hub and hub-hub genes of this module in MAP infection has been demonstrated such as AP1B1, LAPTM4B, MYO1C, ADCY7 (Marino et al., 2017), TNFAIP8L1, MAPK8IP1 (Mackintosh, 2012), and SLC11A1 (Ruiz-Larrañaga et al., 2010). For instance, SLC11A1 (hub gene) plays an essential role in innate immune and prevention of bacterial growth in macrophages during the early stages of infection (Paixão et al., 2007). Accordingly, Ruiz-Larrañaga et al. (2010) detected a significant genetic association between two SNPs in the bovine SLC11A1 gene and susceptibility to infection by MAP in Holstein-Friesian cattle (Ruiz-Larrañaga et al., 2010). MAPK8IP1 (hub-hub gene), reported to be upregulated in MAP-infected animals, is related to apoptosis and autophagy. In fact, destroying macrophages through apoptosis and autophagy is a key defense mechanism against MAP (Mackintosh, 2012). MAPK8IP1 is one of the mitogen-activated protein kinase (MAPK) signaling cascade genes which is involved in the activation of downstream cellular responses upon the recognition of mycobacterial pathogen-associated molecular patterns (PAMPs) by cell surface pathogen recognition receptors (PRRs), such as the Toll-like receptors (TLRs) and the receptor tyrosine kinases (RTKs) (Weiss and Souza, 2008). AP1B1 (hub gene) encodes lysosomal enzymes and its expression decreases during MAP infection. LAPTM4B (hub gene) is a lysosomal membrane protein and ACP5 (hub gene) encodes sulfatase lysosomal enzyme, and both genes show downregulation (Marino et al., 2017). Suppression of lysosomal function may be a general defense mechanism used by mycobacteria to increase survival following phagocytosis by macrophages (Podinovskaia et al., 2013). ADCY7 and MYO1C (hub genes) both were overrepresented in monocyte-derived macrophages infected with MAP and are involved in the immune system (Marino et al., 2017). In terms of lncRNA, three genes were found in the green module as both cis and trans targets of lncRNAs including KIAA1671 in upstream of ENSBTAG00000053952 lncRNA, KIAA0753 in downstream of ENSBTAG00000051146 lncRNA and SH3BP4 (hub gene) in downstream of ENSBTAG00000053153 lncRNA. Interestingly, ENSBTAG00000053153 lncRNA was also a hub-hub gene. KIAA0753 has protein-binding function and encodes a protein that regulates centriolar duplication. SH3BP4 is involved in cargo-specific control of clathrin-mediated endocytosis, specifically controlling the internalization of a specific protein receptor (O’Leary et al., 2016).

### Royalblue Module

The royalblue module illustrated that their genes were enriched in some important biological processes related to MAP infection such as “regulation of endoplasmic reticulum stress-induced intrinsic apoptotic signaling pathway,” “defense response,” “neutrophil activation,” and “neutrophil chemotaxis.” MAP influences cell survival by disrupting the immune response in infected macrophages. In fact, MAP inhibits the apoptotic pathways in order to support host cell survival (Behar et al., 2010). Neutrophils are usually employed to disease lesions, caused by MAP infection, in the gut (Khare et al., 2009). It is indicated that the inflammation in the ileocecal valve is intensified in the clinical animals by an increased recruitment of neutrophils (Hempel et al., 2016). In the royalblue module, several hub genes have been previously reported to be in association with MAP infection. IFNGR1 (hub gene) is an IFN-γ receptor with expression decreases in MAP-infected macrophages (Marino et al., 2017). Considering the importance of IFN-γ in the control and elimination of intracellular pathogens, it seems that it is a necessary defensive mechanism of the host which MAP tries to escape (Arsenault et al., 2014). IL10RA (hub gene) encodes the ligand-binding subunit of the IL10R and is an important factor for IL-10 responsiveness (Ding et al., 2001; Tamassia et al., 2008). IL-10 is an immunoregulatory cytokine produced by macrophages and other immune cells that inhibits the antimicrobial activity of the macrophage (Romano et al., 1996). In the royalblue module, CDH26 was detected as both cis and trans target of lncRNA ENSBTAG00000050877. This lncRNA was a hub-hub gene, and its target gene encodes a member of the cadherin protein family. The protein is expressed in gastrointestinal epithelial cells and may be upregulated during allergic inflammation. Moreover, this protein interacts with alpha integrins and may also be involved in leukocyte migration and adhesion (O’Leary et al., 2016).

### Purple Module

Similar to the other modules, this module was also observed to be related to MAP infection in terms of functional enrichment of its genes. Some important enriched terms were “cellular response to interleukin-4,” “regulation of T cell proliferation,” “T cell differentiation,” “regulation of leukocyte cell-cell adhesion,” “positive regulation of lymphocyte activation,” and “leukocyte activation involved in immune response.” As infected cells migrate to local lymphoid tissue, interactions between lymphocytes and activated antigen presenting cells start immune responses. Confronting antigens leads specific T cells to proliferate and differentiate into effector cells. The cell-mediated immune response of host is crucial to battle against mycobacterial disease (Clarke, 1997; Pollock et al., 2001; de Silva et al., 2010). Since MAP remains within macrophages and other infected cells, the host immune system organizes a prolonged immune response with activated cytotoxic T cells, γδ T cells, CD4 cells, and cytokines, leading to granuloma formation and diffusion of the infection (Coussens, 2001). γδ T cells are the main subset of circulating lymphocytes in ruminants, especially in young animals, which are most susceptible to JD (Larsen et al., 1975). The proliferation of regulatory T cells is suggested to be a potential mechanism to suppress the adaptive immune response in the MAP-infected animals (Weiss et al., 2006). Intramucosal leukocyte accumulation along with the increase of other immune system cell activity in MAP-exposed mice is suggested to indicate part of the host immune response to MAP (Roderfeld et al., 2012). IL-4 is a multifunctional cytokine produced by CD4+ Th2 cells, basophils, and mast cells which is important for granuloma formation in mycobacterial infection and defense against mycobacterial infection (Sugawara et al., 2000). Some of the related genes to MAP infection in this module based on the previous reports are as follows: CORO1A (Ariel et al., 2019), CD81 (Marino et al., 2017), and IRF1 (Machugh et al., 2012; Marino et al., 2017). CORO1A (hub gene) has different functions in calcium homeostasis, cell skeletal dynamics, and maintaining the diversity and function of immune cells (Jayachandran and Pieters, 2015). CORO1A is present in the phagosomal membrane and prevents lysosomal fusion. In mycobacterium-infected cells, inhibition of fusion between phagosomes and lysosomes contributes to mycobacterial survival (Ariel et al., 2019). IRF1 is a member of the family of interferon-regulating transcription factors and is involved in the immune system (Marino et al., 2017). This gene activates interferon-alpha and beta transcription (Machugh et al., 2012). IRF1 is an important transcription factor involved in type 1 cell-dependent immune response (Th1) and regulates the expression of many genes involved in phagocytosis. Cellular immunity is an important host-defense mechanism against intracellular pathogens such as MAP (Koets et al., 2002).

### Darkturquoise Module

The most significant GO terms enriched in the darkturquoise module were “complement activation; classical pathway” and “positive regulation of wound healing.” The complement receptor is used by the pathogenic mycobacteria to enter macrophages (Seth et al., 2009). *Mycobacterium tuberculosis*-specific antibodies increase complement activation through both classical and alternative pathways, so phagocytosis of antibody-opsonized bacteria by macrophages is intensified (Lande et al., 2003). The downregulation of various complement associated genes due to MAP infection proposed that the complement system is inhibited to some extent by macrophage infection, which may support MAP survival (Marino et al., 2017). Investigation of relationships of darkturquoise module hub genes with MAP infection was indicative of the association of many genes with the disease such as BOLA-DMB, BOLA-DMA, C1QA, C1QB, C1QC (Marino et al., 2017), BLA-DQB (Mackintosh, 2012). BOLA-DMB, and BOLA-DMA (hub-hub genes) are key regulators of MHC class II antigen presentation to T cells (Pos et al., 2013; Mellins and Stern, 2014). BLA-DQB (hub-hub gene) is one of the MHC class II genes and is prohibited in infected animals. Inhibition of these genes potentially influences on the ability of host to present MAP peptide fragments of endocytosis to CD4+T lymphocytes (Mackintosh, 2012). MHC class II molecules are involved in the development of a humoral immune response to MAP although MHC class I molecules are responsible for T toxic cell detection in infected cells. Accordingly, MAP may affect the type and specificity of host immune response (Marino et al., 2017). CD4+, CD8+, and γδ subsets of T cells in protective immunity against mycobacterial pathogens predominantly function as a source of cytokines which either directly activates macrophages to kill mycobacteria or participate in the extension of other T cells involved in infection (Stabel, 2000). MAP infection has a potential influence on the trafficking and migration of phagocytes to the site of inflammation. CMPK2 (hub gene) is one of the genes responsible for the response to type 1 interferon. The upregulation of CMPK2 concurrent with MAP infection is indicative of the role of type 1 interferon signaling in MAP-macrophage interaction (Gupta et al., 2019). HMGCR (hub gene) is a lipid biosynthesis gene and its expression increases by MAP (Ariel et al., 2019; Johansen et al., 2019). This gene has a protective effect on the cholesterol-filled lysosomal membrane (Ariel et al., 2019). In fact, MAP focuses on cholesterol-rich parts in macrophages and causes impairment in phagosome maturation (Huynh et al., 2008; Keown et al., 2012).

### White Module

The white module was found to be predominantly enriched in “phagocytosis,” “T-cell migration,” “neutrophil chemotaxis,” “defense response,” and “defense response to bacterium.” Immune response-related genes in the white module indicated that these genes are likely related to MAP infection. Furthermore, in this module, there are many hub-hub, hub, and TF genes which were previously presented to have a tendency to affect MAP infection such as ISG15 (Mackintosh, 2012), LDLR (Ariel et al., 2019; Johansen et al., 2019), TIMD4, MAP2K6, MAOA, F13A1 (Marino et al., 2017), PRMT1, TREM1 (Ariel et al., 2019), PRKCI (Kabara et al., 2010), and SAA3 (Machugh et al., 2012; Casey et al., 2015). Notably, F13A1 (hub-hub gene) encodes coagulation factor XIIIA and is reported to be differentially transcribed in monocyte-derived macrophages infected with MAP (Marino et al., 2017). LDLR (hub-hub gene) takes part in cholesterol homeostasis (Thirunavukkarasu et al., 2014). When intracellular cholesterol levels are high in host macrophages due to mycobacterial infection (De Chastellier and Thilo, 2006), cholesterol biosynthesis and transport are blocked by a feedback mechanism (Brown and Goldstein, 1986). Reducing intracellular cholesterol favors apoptosis and prevents cholesterol-induced obstruction in phagosome maturation, which inhibits pathogen survival (Thirunavukkarasu et al., 2014). This gene is one of the genes involved in lipid endocytosis, which has been highly expressed in MAP-infected macrophages (Johansen et al., 2019). MAP increases the uptake of LDL and modified LDL by increasing the expression of receptors such as LDLR (Ariel et al., 2019). TREM1 (hub gene) is involved in inflammatory signaling pathways (Ariel et al., 2019). This gene is a potent enhancer of inflammatory response to invading microbes (Lagler et al., 2009) and may act as a key player in innate immunity during MAP infection (Ariel et al., 2019). SAA3 (hub gene) has an immune function (Machugh et al., 2012) and is one of the genes encoding proteins associated with the induction and activation of inflammasome. SAA3 gene encodes an important protein in the acute phase of macrophages (Ather et al., 2011).

On the other hand, some of the predicted target genes of the lncRNAs in the nonpreserved modules revealed important information related to *in vitro* MAP infection in macrophages. In this regard, a cis target genes was found in the salmon module called BOLA-DMA (Marino et al., 2017) in upstream of lncRNA ENSBTAG00000052980. It is worth noting that this lncRNA (ENSBTAG00000052980) was a hub-hub gene. The importance of BOLA-DMA as a key regulator of MHC class II antigen presentation to T cells was discussed above (Pos et al., 2013; Mellins and Stern, 2014). Furthermore, TSHZ1 gene (hub gene and TF) in the salmon module was considered as both cis and trans targets of lncRNA ENSBTAG00000049661. TSHZ1 encodes a colon cancer antigen in humans, and the encoded protein may be involved in the transcriptional regulation of developmental processes (O’Leary et al., 2016). In the darkgrey module, some of the lncRNAs regulated cis targets related to MAP infection including PRKCI (Kabara et al., 2010), a cis target gene in the downstream of lncRNA ENSBTAG00000049676 and TFRC (Marino et al., 2017), a cis target gene in the downstream of lncRNA ENSBTAG00000049459. It should be noted that ENSBTAG00000049676 and ENSBTAG00000049459 lncRNAs were hub-hub genes. PRKCI functions in apoptosis regulation (Kabara et al., 2010). TFRC is one of the key elements of iron metabolism, which transfers iron from transferrin protein to cells. In fact, iron is an important nutrient factor in the innate immune response to a bacterial pathogen (Johnson and Wessling-Resnick, 2012).

These findings make the mentioned modules as well as their members, especially hub and hub-hub genes, as the most important candidates in MAP infection development, and they can be considered to increase our knowledge of the molecular mechanisms involved in MAP-macrophage interaction. Although our results allowed us to have a better comprehension on the potential molecular mechanisms underlying MAP infection which can lead us to recognize JD pathogenesis, further researches still need to be performed to understand the exact biological function of the proposed candidate modules and genes.

## Conclusion

Regarding the considerable impact of JD on the global economy and the limitations of early detection of this disease due to delayed onset of clinical symptoms, a system biology approach was applied to elucidate the molecular networks involved. Many hub and hub-hub genes with related biological functions to MAP infection were identified in the nonpreserved modules that seem to be involved in this disease. As expected, some of these genes were reported to be associated with MAP infection. These hub and hub-hub genes are likely key gene regulators of the module and thereby are considered important for the pathogenesis of MAP. Furthermore, several hub and hub-hub lncRNAs and TFs were identified in the non-preserved modules. The importance of lncRNAs in the regulatory mechanism of inflammatory and immune responses is highlighted in a number of studies (Marques-Rocha et al., 2015; Fernandes et al., 2019). Moreover, it is claimed that lncRNAs have major superiorities as diagnostic and prognostic biomarkers compared with mRNAs (Hauptman and Glavač, 2013). Accordingly, lncRNAs and their cis/trans targets in the non-preserved modules have high potential to be biomarkers of MAP infection. From another side, if intra-modular hub genes are TFs or targets of a TF, this TF is more likely to have a causal role in the phenotype under investigation (Hudson et al., 2009). Correspondingly, the hub and hub-hub TF genes can be considered as regulatory elements involved in the host response to MAP infection, which modulate mRNA genes of the respective modules. To sum up, the identified genes in the current study can serve as promising targets for precise diagnosis of MAP infection and may help to identify the pathways associated with JD as well.

## Data Availability Statement

RNA-Seq data of healthy and infected bovine samples were obtained from the Gene Expression Omnibus (GEO) database at the National Center for Biotechnology Information (NCBI) under accession number of GSE62048.

## Author Contributions

MB, FD, and MH conceived the ideas. MB designed the study. MH and MB analyzed the data. MH interpreted the data and wrote the main manuscript text. AP supervised this work and provided financial support for the project. MB, AP, and FD reviewed and edited the manuscript. All authors read and approved the final manuscript.

## Conflict of Interest

The authors declare that the research was conducted in the absence of any commercial or financial relationships that could be construed as a potential conflict of interest.

## Supplementary Material

Supplementary File 1 Soft threshold selection process used to obtain the sale free topology index.

Supplementary File 2 Descriptive statistics of sequence quality and mapping rate of the RNA-Seq data.

Supplementary File 3 The gene expression matrix of all samples in normal and MAP-infected groups.

Supplementary File 4 The list of genes in each module.

Supplementary File 5 The complete list of the hub genes in each module.

Supplementary File 6 Summary of module preservation analysis.

Supplementary File 7 Enrichment analysis in the preserved modules. Visualization of functional enrichment analysis in clusters in the preserved modules.

Supplementary File 8 The complete list of the functional enrichment analysis of the genes in the preserved modules.

Supplementary File 9 Enrichment analysis in the non-preserved modules. Visualization of functional enrichment analysis in clusters in the nonpreserved modules.

Supplementary File 10 Biological processes of GO analysis for genes in the non-preserved modules of interest.

Supplementary File 11 The complete list of the functional enrichment analysis of the genes in the nonpreserved modules.

Supplementary File 12 The complete list of transcription factors per module.

Supplementary File 13 The complete list of lncRNAs per module.

Supplementary File 14 The complete list of lncRNAs cis targets per module.

Supplementary File 15 The complete list of the both cis and trans targets of lncRNAs in some modules.

Supplementary File 16 The mRNA-lncRNA-TF interaction networks in the nonpreserved modules.

Supplementary File 17 The complete list of the hub-hub genes in nonpreserved modules.

Supplementary File 18 The overview of the non-preserved modules which had significant terms associated with MAP infection and JD.

## Abbreviations

GO: gene ontology
JD: Johne’s disease
lncRNAs: long noncoding RNAs
MAP: mycobacterium avium subsp. paratuberculosis
PPI: protein-protein interaction
TFs: transcription factors
TOM: topological overlap matrix
WGCNA: weighted gene coexpression network analysis.
