# Supplementary material for: Integrated Analysis of lncRNAs, mRNAs, and TFs to Identify Regulatory Networks Underlying MAP Infection in Cattle
Source: Front Genet. 2021 Jul 5;12:668448. doi: 10.3389/fgene.2021.668448 (PMC8287970; doi:10.3389/fgene.2021.668448)
Supplement: Supplementary File 1 — Soft threshold selection process used to obtain the sale free topology index. [file Data_Sheet_1.zip › Data Sheet 1/Supplementary_Materials/Supplementary_File_S16.docx]

**Supplementary File S16:** The mRNA-lncRNA-TF interaction networks in the non-preserved modules.

**Black module**

**Darkgrey module**

**Darkolivegreen module**

**Darkred module**

**Darkturquoise module**

**Green module**

**Grey60 module**

**Lightcyan module**

**Lightgreen module**

**Lightyellow module**

**Orangered4 module**

**Paleturquoise module**

**Plum1 module**

**Purple module**

**Royalblue module**

**Saddlebrown module**

**Salmon module**

**Skyblue3 module**

**Tan module**

**White module**

**Yellow module**
