# Supplementary material for: Integrated Analysis of lncRNAs, mRNAs, and TFs to Identify Regulatory Networks Underlying MAP Infection in Cattle
Source: Front Genet. 2021 Jul 5;12:668448. doi: 10.3389/fgene.2021.668448 (PMC8287970; doi:10.3389/fgene.2021.668448)
Supplement: Supplementary File 1 — Soft threshold selection process used to obtain the sale free topology index. [file Data_Sheet_1.zip › Data Sheet 1/Supplementary_Materials/Supplementary_File_S18.docx]

Supplementary File S18: The overview of the non-preserved modules which had significant terms associated with MAP infection and JD.

**Black module**

Functional enrichment showed that the genes in the black module were overrepresented in “positive regulation of intrinsic apoptotic signaling pathway,” and “response to the toxic substance”. Furthermore, some of the hub genes were associated with JD according to previous studies. BAX is a pro-apoptotic gene and has shown up-regulation in 24 hours after MAP infection compared with the control group (Periasamy, Tripathi, & Singh, 2013). CD59 hub-hub gene is one of the key genes in the hematopoietic cell lineage pathway, so that activation of this pathway is representative of the host immune response to MAP. CD59 regulates complement-mediated cell lysis, has a role in lymphocyte signal transduction, and it is a potent inhibitor of the complement membrane attack complex. In addition, it is involved in signal transduction pathways in the activation of T cells (Khare et al., 2012). It is reported that CD59 has been a differentially transcribed gene in monocyte-derived macrophages infected with MAP (Marino et al., 2017). STX8 hub gene, which prevents host immune response through suppression of microtubule-associated protein 1 pathway, was down-regulated in the late phase of infection (Khare et al., 2012). PARP1 hub gene plays important roles in initiating apoptosis and DNA repair and has been associated with JD in several studies (Kabara et al., 2010; Colin Mackintosh , 2012; Settles et al., 2009). DLD hub gene encodes dihydrolipoamide dehydrogenase enzyme in humans. In cows, differences in DLD genotypes and therefore probable ability to use nutritive can explain why some animals enter the advanced clinical stage of JD while others do not move into this phase despite intestinal damages (Van Hulzen et al., 2012). CD63 hub gene encodes for lysosomal integral membrane proteins and was upregulated in infected animals (Colin Mackintosh, 2012; Smeed, Watkins, Gossner, & Hopkins, 2010). BPNT1 hub gene functions in the biological oxidations pathway. It was reported that DYNC1LI1 hub gene participates in immune-related pathways. BPNT1 and DYNC1LI1 have been overrepresented in monocyte-derived macrophages infected with Mycobacterium (Marino et al., 2017). ASAH1 hub gene takes part in the lysosomal pathway of infection control. It was identified as a differentially expressed gene involved in relevant pathways between clinical and subclinical animals (Hempel, Bannantine, & Stabel, 2016b).

**Green module**

The involvement of many hub genes included in the green module with JD has been demonstrated by several researchers. These genes are as follows: ETS2 (Blumerman, Herzig, Wang, Coussens, & Baldwin, 2007), MAPK8IP1 (Colin Mackintosh, 2012), AP1B1 (Marino et al., 2017), LAPTM4B (Marino et al., 2017), TNFAIP8L1 (Colin Mackintosh, 2012; Pant et al., 2010), SLC11A1 (Pinedo et al., 2009; Ruiz-Larrañaga et al., 2010), ITGA5 (Alluwaimi & Badi, 2015), TTYH2 (Alonso-Hearn et al., 2019; Shin et al., 2015), JSP.1 (Shin et al., 2015), ADCY7 (Marino et al., 2017), MYO1C (Marino et al., 2017), ADIPOR1 (Khare et al., 2012; Mallikarjunappa et al., 2018), TPRG1L (Mallikarjunappa et al., 2018; Shin et al., 2015). VDR (TF gene) is also introduced as a candidate gene associated with susceptibility to JD (McSpadden, Caires, & Zanella, 2013). Different roles such as immune cell trafficking, leukocyte migration, vitamin and mineral metabolism, cell signaling, hematological system development and function were mentioned for VDR (Alluwaimi & Badi, 2015). ETS2 is a transcription factor involved in the regulation of CDKN2A and the prevention of replicative senescence. Generally, ETS2 has a role in cell death (Blumerman et al., 2007). MAPK8IP1 hub-hub gene is one of the genes related to apoptosis and autophagy, which encodes a regulator of the pancreatic beta-cell function (Colin Mackintosh, 2012). MAPK8IP1 hub-hub gene, which has previously showed upregulation in JD infected animals, is related to apoptosis and autophagy. In fact, destroying macrophages through apoptosis and autophagy is a key defense mechanism against MAP (Colin Mackintosh, 2012). MAPK8IP1 is one of the mitogen activated protein kinase (MAPK) signalling cascade genes which is involved in the activation of downstream cellular responses upon the recognition of mycobacterial pathogen-associated molecular patterns (PAMPs) by cell surface pathogen recognition receptors (PRRs), such as the Toll-like receptors (TLRs) and the receptor tyrosine kinases (RTKs) (D. Weiss & Souza, 2008). AP1B1 encodes lysosomal enzymes and its expression decreases during MAP infection. LAPTM4B is a lysosomal membrane protein and ACP5 encodes sulfatase lysosomal enzyme, and both genes show downregulation (Marino et al., 2017). TNFAIP8L1 gene is identified as an important protein in keeping immune homeostasis (Lou & Liu, 2011). Suppression of lysosomal function may be a general defense mechanism used by mycobacteria to increase survival following phagocytosis by macrophages (Podinovskaia, Lee, Caldwell, & Russell, 2013). SLC11A1 encodes natural resistance protein related to macrophages (NRAMP1) and is associated with a natural resistance against intracellular pathogens (Paixão et al., 2007). NRAMP1 has a role in the recycling of hemoglobin-derived iron through phagocytosis (Soe-Lin, Sheftel, Wasyluk, & Ponka, 2008). Furthermore, other researchers reported the associations of SLC11A1 gene with susceptibility to JD in sheep (Reddacliff, Beh, McGregor, & Whittington, 2005). ITGA5 which increases in infected animals functions in immune cell traffic including cell movement and leukocytes migration and also phagocytes cell movement (Alluwaimi & Badi, 2015). The upregulation of TTYH2 in MAP infected cows was reported. TTYH2 contributes to innate immune response Ca2+ signal transduction, cell proliferation, and cell aggregation (Shin et al., 2015). TTYH2 is a paralogue of TTYH3, which is a calcium-dependent chloride channel and has key roles in functions related to innate immune response (Moreland, Davis, Bailey, Nauseef, & Lamb, 2006). JSP.1 gene is also upregulated and participates in antigen-processing, antigen-presenting via MHC class I, and positive regulation of T cell-mediated cytotoxicity (Shin et al., 2015). ADCY7 and MYO1C both were overrepresented in monocyte-derived macrophages infected with MAP and are involved in the immune system (Marino et al., 2017). ADIPOR1 gene is a receptor of ADIPOQ gene, and interaction between this gene and its receptor suppresses the NF-kB-dependent synthesis of tumor necrosis factor and interferon (Khare et al., 2012). TPRG1L takes part in binding identical proteins, and in various compartments such as synaptic vesicle, cytoplasm, and cell junction (Shin et al., 2015).

**Royalblue module**

In this module, several hub genes have been previously indicated to be in association with JD. ALCAM gene is associated with immunological synapses and T cell receptor complex and it was significantly associated with susceptibility/resistance to JD (Brito et al., 2018). TLR4 hub-hub gene is a member of the TLR family that can detect various classes of pathogens and responsible for the coordination of appropriate innate and adaptive responses (Quesniaux et al., 2004; Tianyi Wang, Lafuse, Takeda, Akira, & Zwilling, 2002). TLR4 is involved in the identification of mycobacterial antigens (Quesniaux et al., 2004; D. J. Weiss, Souza, Evanson, Sanders, & Rutherford, 2008; Yadav & Schorey, 2006), cytokine production mediation and host defense stimulation. Interestingly, it has been demonstrated that TLR4 can independently recognize MAP (Ferwerda et al., 2007). Mutation in TLR4 causes an ineffective diagnosis of MAP and failure in immune responses and accordingly increases the risk of mycobacterial infections (Henckaerts et al., 2007). JAM3 (hub-hub gene) functions in the tight junction pathway. Apparently, tight junction plays an important role in the pathogenic difference of host response to different mycobacteria. This pathway becomes intensively activated in the late phase of MAP infection (Khare et al., 2016). ALOX5AP hub gene is one of the key genes related to immunity and inflammation. ALOX5AP along with ALOX5 participates in leukotriene biosynthesis, which is involved in various inflammatory responses (Ström, Strid, & Hammarström, 2012). Downregulation of genes linked to immunity and inflammation decreases the capacity for the proper inflammatory response in MAP infected animals (David, Barkema, & De Buck, 2014). IFNGR1 is an IFN-γ receptor that its expression decreases in MAP infected macrophages (Marino et al., 2017). Considering the importance of IFN-γ in the control and elimination of intracellular pathogens, it seems that it is a necessary defensive mechanism of the host which MAP tries to escape it (Arsenault et al., 2014). RASGRP1 has a role in the T cell receptor pathway and is differentially expressed in Johne’s infected animals (Hempel et al., 2016b). The ORMDL3 gene is one of the genes involved in Crohn's disease in humans and it has been suggested that genes involved in this disease in humans may be susceptible to cause JD (Zanella et al., 2011). NFATC2IP gene is involved in protein synthesis in the number of cytokines and the development and function of the blood system in the number of blood cells. This gene increased during infection (Alluwaimi & Badi, 2015).

**Purple module**

The genes related to JD in this module based on previous studies are as follows: SCAP (Kiser, Neupane, White, & Neibergs, 2018), H1FX (Coussens, Jeffers, & Colvin, 2004), SBF1 (Kabara et al., 2010), RABGGTA (Alluwaimi & Badi, 2015), THAP3 (Colin Mackintosh, 2012). SCAP hub gene is functional in the vesicular membrane and cytoplasmic vesicular membrane (Kiser et al., 2018). CORO1A hub gene has different functions in calcium homeostasis, cell skeletal dynamics, and maintaining the diversity and function of immune cells (Jayachandran & Pieters, 2015). This gene encodes the protein chronin 1 in mammalian cells and is involved in actin dynamics (Rybakin & Clemen, 2005). Research has shown that chronin 1 is an essential factor in regulating calcium signaling after the invasion of pathogenic mycobacteria. Chronin 1 regulates the flow of physiological calcium ions, induces calcium neurin activation and ultimately inhibits phagosome-lysosome fusion (Jayachandran et al., 2007). In mycobacterium-infected cells, inhibition of fusion between phagosomes and lysosomes contributes to mycobacterial survival (Ariel et al., 2019). BATF2, a member of activating protein 1 transcription factors family (AP-1), interacts with IFN regulatory factor 1 to mediate downstream pro-inflammatory immune responses. This gene is often expressed in monocytes and macrophages and plays a key role in macrophage activation by regulating inflammatory responses during mycobacterial infection (Alonso-Hearn et al., 2019). H1FX hub gene is a replication-dependent histone involved in DNA fragmentation during apoptosis (Coussens et al., 2004). SBF1 hub gene takes part in preventing dephosphorylation (Kabara et al., 2010). RABGGTA hub gene has a role in the metabolic and biosynthetic processes of lipoproteins (Alluwaimi & Badi, 2015). THAP3 TF gene plays role in apoptotic and autophagic processes. This gene is part of the THAP1/THAP3-HCFC1-OGT complex, which is required to regulate RRM1 transcriptional activity (Colin Mackintosh, 2012).

**Darkturquoise module**

Investigation of relationships of darkturquoise module hub genes with JD was indicative of the association of many hub genes with the disease including CTSC (Marino et al., 2017), CREM (Hossain, Tchatalbachev, & Chakraborty, 2006), YARS (David, Barkema, Mortier, Ghosh, & De Buck, 2014), CMPK2 (P. Gupta et al., 2019), BOLA (David, Barkema, & De Buck, 2014), ITGB5 (Marino et al., 2017), PROS1 (Marino et al., 2017), IGFBP6 (Coussens et al., 2005), HMGCR (Ariel et al., 2019; Johansen, de Silva, Plain, Whittington, & Purdie, 2019; Thirunavukkarasu et al., 2014). CTSC hub-hub gene encodes lysosomal proteases. CD4+ CD25+ T cells are important in terms of disease susceptibility (D. Weiss, Evanson, & Souza, 2006). Potent proliferative response of CD4 T cell has been observed in MAP infected animals that is described by upregulation of MHC class II and CD25+ CD4+ genes (Wu et al., 2007)). Downregulation of MHC class II genes has been reported in the blood of calves exposing to MAP, while the expression of MHC class I genes had been increased (Purdie, Plain, Begg, de Silva, & Whittington, 2012). MHC class II molecules are involved in the development of a humoral immune response to MAP; though MHC class I molecules are responsible for T toxic cell detection in infected cells. Accordingly, MAP may effect on type and specificity of host immune response (Marino et al., 2017). CD4+, CD8+ and γδ subsets of T cells in protective immunity against mycobacterial pathogens predominantly function as a source of cytokines which either directly activates macrophages to kill mycobacteria or participate in the extension of other T cells involved in infection (Stabel, 2000). It seems that CD4+ T cell is a source of gamma interferon primary cell response during infection (Bassey & Collins, 1997). CREM TF gene has a pro-inflammatory function and is one of the cAMP-response-element binders (Hossain et al., 2006). YARS hub-hub gene is involved in the synthesis of enzyme tyrosyl-tRNA synthetase. During inflammation, tyrosyl-tRNA synthetase is cleaved into 2 fragments including mini-tyRS and C-tyRS. Mini-tyRS takes part in angiogenesis and these fragments also have a role in the trafficking of phagocytes (Ewalt & Schimmel, 2002). MAP infection has a potential influence on the trafficking and migration of phagocytes to the site of inflammation. CMPK2 is one of the genes responsible for the response to type 1 interferon. The upregulation of CMPK2 concurrent with MAP infection is indicative of the role of type 1 interferon signaling in MAP-macrophage interaction (P. Gupta et al., 2019). BOLA is a key antigen-presenting protein that carries intracellular proteins to the cellular membrane and presents them to toxic T cells in order to diagnose and final control or killing the intracellular pathogens (David, Barkema, & De Buck, 2014). ITGB5 in phagocytosis, PROS1 in the immune system, and complement cascade (Marino et al., 2017), and IGFBP6 in signal transduction (Coussens et al., 2005) have roles. HMGCR takes part in cholesterol biosynthesis (Thirunavukkarasu et al., 2014) and encodes Cholesterol synthesis restriction enzyme (Johansen et al., 2019). HMGCR expression increases by MAP and has a protective effect on the cholesterol-filled lysosomal membrane (Ariel et al., 2019). In fact, MAP focuses on cholesterol-rich parts in macrophages and causes impairment in phagosome maturation (Huynh, Gershenzon, & Grinstein, 2008; Keown, Collings, & Keenan, 2012).

**White module**

As demonstrated by other researchers, many hub genes were related to JD in this module including ISG15 (Colin Mackintosh, 2012; Mallikarjunappa et al., 2018), CARD11 (Hempel et al., 2016b), LDLR (Ariel et al., 2019; Johansen et al., 2019; Thirunavukkarasu et al., 2014), TIMD4 (Alpay et al., 2014; Gao et al., 2018; Marino et al., 2017), PRMT1, TREM1 (Ariel et al., 2019), PRKCI (Kabara et al., 2010), SAA3 (Casey et al., 2015; MacHugh et al., 2012), MAP2K6 (Kiser et al., 2018; Marino et al., 2017), ENPP2 (Alonso-Hearn et al., 2019), F13A1 (Marino et al., 2017; McGovern et al., 2019), DAB2 (Shin et al., 2015), MAOA (Marino et al., 2017), TCF7 (Gossner, Watkins, Chianini, & Hopkins, 2017). ISG15 hub-hub gene, which is linked to the immune response to invasive pathogens (Colin Mackintosh, 2012), is a ubiquitin-like protein that conjugates to many cellular proteins after activation by interferon alpha and beta (Zhao, Denison, Huibregtse, Gygi, & Krug, 2005). CARD11 gene is involved in the T cell receptor and B cell receptor pathways (Hempel et al., 2016b). LDLR hub-hub gene takes part in cholesterol homeostasis and decreased expression of this gene affects cholesterol transfer (Thirunavukkarasu et al., 2014). Inflammation with lipopolysaccharides increases host serum cholesterol levels by reducing the expression of genes such as LDLR, which play a key role in the absorption of low-density lipoproteins (Yoo & Desiderio, 2003). When intracellular cholesterol levels are high in host macrophages due to mycobacterial infection (De Chastellier & Thilo, 2006), cholesterol biosynthesis and transport are blocked by a feedback mechanism (Brown & Goldstein, 1986). Reducing intracellular cholesterol favors apoptosis and prevents cholesterol-induced obstruction in phagosome maturation, which inhibits pathogen survival (Thirunavukkarasu et al., 2014). This gene is one of the genes involved in lipid endocytosis, which has been highly expressed in another study in MAP-infected macrophages (Johansen et al., 2019). MAP increases the uptake of LDL and modified LDL by increasing the expression of receptors such as LDLR (Ariel et al., 2019). TIMD4 gene plays role in regulating T cell proliferation (Meyers et al., 2005). The activity of PRMT1 gene is essential for the transcriptional function of c-Myc proto-oncogene in the differentiation of M2 macrophages (Tikhanovich et al., 2017). PRKCI gene has a role in the regulation of apoptosis (Kabara et al., 2010). MAP2K6 gene is involved in the immune system pathways, interleukin signaling, cytokine signaling (Marino et al., 2017), and the Toll pathway (Kiser et al., 2018). ENPP2 gene takes part in the immune response pathway (Alonso-Hearn et al., 2019). DAB2 hub-hub gene plays role in the immune response and functions in myeloid cell differentiation, macrophage adhesion and proliferation, and tumor suppression (Shin et al., 2015). MAOA gene is involved in biological oxidations (Marino et al., 2017). TCF7 TF gene is a major regulator of T cell maturation and intrinsic lymphoid cells (Gossner et al., 2017).

**Darkolivegreen module**

According to functional enrichment analysis, the genes of the darkolivegreen module were enriched in “defense response to bacterium,” and “negative regulation of neuron apoptotic process”. The involvement of many hub genes included in this module with JD has been demonstrated by several researchers: SNX24 (Kiser et al., 2018), RNASE6 (Dobson, 2012), PPT1 (Marino et al., 2017), NLRC4 (MacHugh et al., 2012), CFLAR (MacHugh et al., 2012), CDC42BPA (Mallikarjunappa et al., 2018). SNX24 gene is one of the vesicle membrane genes and has an important role in the immune system (Kiser et al., 2018). Vesicles play an important role in the immune system, so that they moderate immune cell stimulation, and also affect inflammatory responses via the transfer of various proteins and RNAs (Robbins & Morelli, 2014). After infection, MAP first invades macrophages through phagocytosis, where it survives by preventing proper phagosome-lysosome fusion using an unknown mechanism (Woo, Heintz, Albrecht, Barletta, & Czuprynski, 2007). The fusion of the phagosome with the lysosome is a necessary step for phagosome–lysosome maturation and finally killing the ingested bacteria (Luzio, Pryor, & Bright, 2007). Vesicles also take part in NF-κB activation through the toll pathway, as vesicle membrane TLRs can stimulate NF-κB activation (Kawai & Akira, 2007). RNASE6 gene has been expressed in neutrophils, monocytes, and macrophages. This gene, generally, participates in the immune system and defense mechanism. PPT1 is one of the genes encoding lysosomal enzymes and is downregulated as a result of MAP infection in monocyte-derived macrophages (Marino et al., 2017). The suppression of lysosomal function may be a general defense mechanism used by mycobacteria to promote their survival following phagocytosis by macrophage (Podinovskaia et al., 2013). NLRC4 gene is a subset of NLRs that takes part in the creation of inflammation and causes activation of caspase-1 and maturation of IL-1ꞵ (G. Chen, Shaw, Kim, & Nuñez, 2009). This gene, also, inhibits autophagy and induces pyroptosis which causes a better inflammatory response (Suzuki et al., 2007). CFLAR gene encodes for proteins whose isoforms act as pro/anti-apoptotic mediators (MacHugh et al., 2012). CDC42BPA gene plays an important role in immunological processes (Mallikarjunappa et al., 2018). This gene is related to MAP entrance to host cells and initiation of immune responses due to MAP infection through regulation of GTPases. Improving the function of GTPases can affect host sensitivity by altering the pathways that MAP uses to cross the intestinal epithelium and infect the host (Kiser et al., 2017).

**Darkred module**

The darkred module was found to be predominantly enriched in “positive regulation of B cell differentiation,” “regulation of activated T cell proliferation,” “regulation of cell-cell adhesion,” and “positive regulation of myeloid leukocyte mediated immunity”. Significant enrichment of many immune response-related genes in the darkred module revealed that these genes are likely related to JD. Furthermore, in this module there are some hub-hub, hub, and TF genes which are previously presented to have a tendency to affect JD such as BCL2L1 (McGarvey, Wagner, & Bermudez, 2004), TOB2 (Kabara et al., 2010), PIAS4 (Colin Mackintosh, 2012), MMP14 (Coussens et al., 2005; David, Barkema, & De Buck, 2014; Malvisi et al., 2016), TICAM1 (Pant et al., 2010), ABL1 (Alluwaimi & Badi, 2015; David, Barkema, Mortier, et al., 2014), IRF5 (Park, Park, Jung, & Yoo, 2018). BCL2L1 hub-hub gene encodes an apoptosis factor that prevents apoptosis in many types of cells (McGarvey et al., 2004). TOB2 hub-hub gene functions in the regulation of cell cycle progression. This gene showed altered expression in MAP infection (Kabara et al., 2010). PIAS4, a hub TF, is also linked with apoptosis and autophagy and has regulatory roles in various cellular pathways such as the STAT pathway, p53 pathway, Wnt pathway, and steroid hormone signaling pathway (Colin Mackintosh, 2012). MMP14 hub gene, which was significantly enriched in positive regulation of B cell differentiation in the present study, is a member of matrix metalloproteinases encoding genes and their inhibitors (Coussens et al., 2005). Furthermore, MMP14 functions in granulocyte adhesion and diapedesis and was differentially expressed in MAP infected animals (David, Barkema, & De Buck, 2014; Malvisi et al., 2016). According to our GO results, TICAM1 showed significant enrichment in positive regulation of myeloid leukocyte mediated immunity. TICAM1 is a toll-like receptor adaptor molecule which induces type I interferons to function in the immune response against mycobacteria (Lande et al., 2003; Seya, Oshiumi, Sasai, Akazawa, & Matsumoto, 2005) and is associated with JD (Pant et al., 2010). ABL1 hub-hub is an anti-apoptotic gene (David, Barkema, Mortier, et al., 2014), and participates in phagocytes’ cell movement and leukocytes migration (Alluwaimi & Badi, 2015). IRF5, a TF gene, is an interferon regulatory factor and regulates immune response to intracellular pathogens (Park et al., 2018). IRF5 along with IRF7 is involved in the activation of type 1 interferons. Alpha and beta interferons are widely expressed in type 1interferon which has various impacts on innate and adaptive immunity (Honda & Taniguchi, 2006; McNab, Mayer-Barber, Sher, Wack, & O'garra, 2015). The upregulation of these two genes causes activation of alpha/beta interferon signaling during the subclinical stage of JD (Park et al., 2018).

**Yellow module**

Based on GO results, the genes of the yellow module were enriched in different biological processes including “cytokine-mediated signaling pathway,” “defense response to a gram-positive bacterium,” and “acute inflammatory response”. In the yellow module, many hub genes revealed an association with JD in former investigations such as NLRP3 (Ariel et al., 2019; Mallikarjunappa et al., 2018; Marino et al., 2017), PSMD5 (Marino et al., 2017), TRIM13 (David, Barkema, Mortier, et al., 2014), CTNNB1 (Khare et al., 2016; Khare et al., 2012), TLR7 (David, Barkema, Mortier, et al., 2014), GALC (Marino et al., 2017), GPR183 (Shin et al., 2015), TMEM30A (Kiser et al., 2018), BIRC3 (MacHugh et al., 2012), FUCA1 (Malvisi et al., 2016; Marino et al., 2017), MAPK14 (David, Barkema, Mortier, et al., 2014; MacHugh et al., 2012), PPARA (Khare et al., 2012; Kiser et al., 2018). NLRP3 is one of the effective genes in the immune system (Marino et al., 2017). In fact, this gene is a Nod-like intracellular innate immune receptor that recognizes molecular patterns related to pathogens and initiates the induction of pro-inflammatory cytokines such as IL-18 and IL-1ꞵ via activation of caspase-1 in inflammasome complex (Zaki, Lamkanfi, & Kanneganti, 2011). CXCR4 which has been reported to be differentially expressed in infected animals takes part in chemokine receptors binding chemokines (Colin Mackintosh, 2012; Marino et al., 2017). PSMD5 hub-hub gene and TRIM13 hub gene are the genes involved in the immune system (David, Barkema, Mortier, et al., 2014; Marino et al., 2017). CTNNB1 hub-hub gene is a member of the tight junction pathway (Khare et al., 2016). CTNNB1 encodes a protein required for the creation and maintenance of epithelial cell layers by regulating cell growth and adhesion between cells and also supports the actin cytoskeleton (Khare et al., 2012). TLR7 is an intracellular PRR receptor that plays a role in the regulation of autophagy in the cell cytoplasm as a part of the innate defense mechanism (Delgado, Elmaoued, Davis, Kyei, & Deretic, 2008). It seems that autophagy can be activated in MAP infected animals (David, Barkema, Mortier, et al., 2014). GALC gene is located in the lysosomal portion and is part of glycosidases (Marino et al., 2017). GPR183 contributes to correct localization of B cells during humoral immune responses and increases B-cell localization in the outer follicle and interfollicular regions (Shin et al., 2015). TMEM30A gene is involved in the vesicular membrane and cytoplasmic vesicular membrane (Kiser et al., 2018). BIRC3 gene has an anti-apoptotic role (MacHugh et al., 2012). FUCA1 gene is associated with inflammatory responses (Malvisi et al., 2016) and is a component of lysosomal glycosidases (Marino et al., 2017). Enhanced phosphorylation of MAPK14 is primarily responsible for increasing IL-10 expression, which is a key mediator of the innate host immune response to Mycobacterium infection (Reiling, Blumenthal, Flad, Ernst, & Ehlers, 2001; Souza, Evanson, & Weiss, 2007). PPARA TF gene has a role in the Toll pathway (Kiser et al., 2018).

**Salmon module**

GO results indicated that STK4 hub gene, and HLX, NR1D1 TF genes were enriched in “regulation of extrinsic apoptotic signaling pathway via death domain receptors,” and “negative regulation of leukocyte activation,” respectively. The salmon module also has some hub genes that were described as JD-related genes in preceding researches. PRDM2, which is identified as both hub-hub and TF in the current study, is a tumor suppressor gene (O'Leary et al., 2016) and is reported to be significantly associated with MAP (Mallikarjunappa et al., 2018). Moreover, SPEN and TRRAP were hub-hub genes and are involved in the host response to MAP infection (Mallikarjunappa et al., 2018; Settles et al., 2009). STX6 is a member of the syntaxin family that functions in intracellular vesicle trafficking through vesicle fusion and exocytosis. Syntaxins are involved in the Microtubule-Associated Protein 1 (M-AP1) pathway. M-AP1 pathway becomes activated in the early phase of the disease, but it is suppressed in the late stages. The conversion of the activation of the M-AP1 pathway may reveal the main mechanism for MAP to escape host immunity. STX6 with decreased expression causes suppression of the M-AP1 pathway in the late stages of the disease (Khare et al., 2012). EP300 hub-hub gene was involved in the host response to MAP infection and in regulating gene transcription through chromatin regeneration (Ibeagha-Awemu, Do, Dudemaine, & Bissonnette, 2018). ACTN4 plays a role in the positive regulation of cell movement and was introduced as a candidate gene for JD (Neibergs, Settles, Whitlock, & Taylor, 2010). ADAR gene expression increases after inflammation and encodes the enzyme responsible for RNA editing. This enzyme destabilizes double-stranded RNA by converting adenosine to inosine, which alters the viral RNA genome (Colin Mackintosh, 2012). PFKFB3 is known to promote glycolysis and contribute to endothelial inflammation induced by TNF-α (R. Zhang, Li, Liu, Li, & Tang, 2019). PFKFB3 shows upregulation in 4 and 8 hours after infection (Ariel et al., 2019). BCL6 TF gene is a regulator of B cell maturation, memory activation, and development and was repressed during infection (Gossner et al., 2017).

**Orangered4 module**

According to functional enrichment results, the genes in orangered4 were significantly enriched in the positive regulation of myeloid leukocyte differentiation. It was reported by other researches that some of the hub genes of this module were associated with JD. PLCD1 hub-hub gene, which is highly expressed in the early stages of JD, is involved in the pathways of the phosphatidylinositol signaling system and calcium signaling. This gene encodes phosphoinositide-specific phospholipase C, which acts as a signal transmitter and has various biological functions including roles in inflammation, cell growth, signaling and death, and maintenance of membrane phospholipids (Khare et al., 2012). PDK4 is one of the genes with differential expression in MAP-infected animals that is associated with inhibition of functions in lipid metabolism and small molecule biochemistry (Shin et al., 2015). INPP5B gene is related to lipid metabolism and its expression increased during infection (Alluwaimi & Badi, 2015). PDCD4 gene was first identified in the apoptosis process and appears to be involved in tumorigenesis by regulating apoptosis (Z. Chen et al., 2015; Li et al., 2016). Also, this gene has been reported to play an important role in various inflammatory diseases (Liang et al., 2016; Sheedy et al., 2010). It is downregulated in infected animals and it has also been suggested that this gene is a mediator in the regulation of macrophage apoptosis (Tianyu Wang, Li, Nelson, & Nabavi, 2019). AP1S1 gene is involved in the immune system, the development of clathrin-derived vesicles, and the development of trans-Golgi network vesicles and was differentially expressed in animals with JD (Marino et al., 2017). OSM gene, which has been reported to have increased expression in MAP-infected monocyte-derived macrophages, is associated with the JAK-STAT pathway. JAK/STAT signaling pathway is the main signaling cascade involved in activating and regulating the host immune response by kinases and growth factors. Signal transmission by IFNγ is associated with this pathway. MAP may alter or even shut down this signaling cascade to increase its survival in macrophages (Marino et al., 2017). KIF3C gene is one of the genes with differential expression in MAP infected monocyte-derived macrophages and plays role in the immune system (Marino et al., 2017). FOS TF gene is one of the immune-related genes that has been differentially expressed in infected animals (Marino et al., 2017). This gene is one of the transcription factors activated by MAPK (MacHugh et al., 2012) that regulates the expression of genes encoding inflammatory chemokines and cytokines (Y. Zhang & Dong, 2007; Y. L. Zhang & Dong, 2005). ZBTB20 TF gene is one of the genes associated with the blood response to MAP. The product of this gene is involved in metal ion binding processes. The fact that MAP cannot internally produce mycobactin (essential for iron transport) makes it a host-dependent intracellular parasite for absorption and metabolism (Clark, Koziczkowski, Radcliff, Carlson, & Ellingson, 2008; McNees, Markesich, Zayyani, & Graham, 2015; Rathnaiah et al., 2017). MAP may use ZBTB20 as a means of providing exogenous iron for growth and proliferation while destroying the host immune system in target cells. ZBTB20 gene is involved in promoting innate immune responses initiated by the toll-like receptor in the host (Liu et al., 2013). This gene may be associated with bovine resistance status to MAP (McGovern et al., 2019). NR4A2 TF gene has a role in the cellular response to hormonal stimulation and was associated with MAP tissue infection (Kiser et al., 2018).

**Saddlebrown module**

Functional enrichment analysis suggested the saddlebrown module was enriched in positive regulation of T cell differentiation. Several hub genes in this module were found related to JD in other researches including ZAP70 (Gao et al., 2018), CD8A (Gossner et al., 2017), CD6 (Gossner et al., 2017), BOLA-DRA (Marino et al., 2017), UBD (CG Mackintosh et al., 2016; Zare, Shook, Collins, & Kirkpatrick, 2014), CCR7 (David, Barkema, & De Buck, 2014), BOLA-DOA (Marino et al., 2017), CD3E (Smeed et al., 2010), GATA3 (McSpadden et al., 2013; Roussey, Oliveira, Langohr, Sledge, & Coussens, 2016; Yagi, Zhu, & Paul, 2011), CD37 (Khare et al., 2016), CXCL9 (Marino et al., 2017; Park et al., 2018), CD3D (Hempel et al., 2016b), ITK (Sallam, Zare, Shook, Collins, & Kirkpatrick, 2018). ZAP70 gene is one of the genes involved in the biological processes of immunity. This gene encodes an enzyme that belongs to the tyrosine kinase protein family involved in T cell development and lymphocyte activation (Gu et al., 2006). This enzyme is associated with T cell receptor and is also involved in the activation of NF-κB in T cells (Weil & Israël, 2004). Changes in the expression levels of this gene are consistent with changes in phosphorylation, which is known to underlie the activation of many pathways in activated lymphocytes (Cannons & Schwartzberg, 2004). Investigation of the expression of this gene in MAP-infected cows by researchers revealed that expression levels of ZAP70 decreased in CD4+ T cells of clinically and subclinically infected animals, indicating a change in T cell phenotype with disease status (Leite et al., 2015). CD8A hub-hub and CD6 hub genes are involved in the T cell pathway and are markers of lymphocyte level (Gossner et al., 2017). BOLA-DRA gene is a classic component of MHC class II whose expression level decreases with MAP infection. Given the key role of MHC class II antigens in focusing on the immune response, decreased expression of MHC class II expression during MAP infection may delay or even inhibit the activation of the immune response against infectious pathogens (Marino et al., 2017). UBD gene, also known as FAT10, alters an inflammatory mediator and limits its activity during the cellular response. In general, FAT10 is involved in the signaling cascades of pro-inflammatory cytokines (Buchsbaum, Bercovich, Ziv, & Ciechanover, 2012). This gene also has a role in the regulation of apoptosis (CG Mackintosh et al., 2016). CCR7 gene belongs to G-protein-coupled receptors present on B and T lymphocytes and leukocytes, enabling these cells to move to infection sites and secondary lymphoid organs (Johnson & Jackson, 2014; Schaeuble, Hauser, Singer, Groettrup, & Legler, 2011). BOLA-DOA gene is one of the key regulators of MHC class II antigen-presenting to T cells (Mellins & Stern, 2014; Pos, Sethi, & Wucherpfennig, 2013). CD3E hub-hub gene encodes elements of the T cell receptor complex (Smeed et al., 2010). GATA3 TF gene is an immune system gene. This gene is a TH2-derived transcription factor that has been highly expressed during infection. CD4+ T cell populations respond to a combined Th1/Th2 response to primary MAP infection (Roussey et al., 2016). GATA3 is responsible for inducing Th2 differentiation and suppresses Th1 differentiation by preventing increased IL12 ꞵ2 receptor expression and signal transducer and transcription activator 4 (STAT4) and neutralizing Runx3 function through protein-protein interactions (Yagi et al., 2011). CD37 gene is one of the genes that play an important role in the activation of T cells and B cells during MAP infection (Khare et al., 2016). CXCL9 gene is a chemokine that has a role in the binding pathway of chemokine receptors to chemokines (Marino et al., 2017). CD3D hub-hub gene is involved in the T cell receptor pathway (Hempel et al., 2016b). ITK hub-hub gene encodes an intracellular tyrosine kinase that is primarily expressed in T cells (Sahu & August, 2009). This gene takes part in T cell development and function, T cell receptor signaling, cytokine release and differentiation regulation, as well as the development of immune cells and multiple inflammatory diseases (Sahu, Mueller, Fischer, & August, 2008). ITK plays a vital role in the regulation of T cell receptors and IL-2 secretion and the regulation of TH2 cytokines and is a key mediator of TCR downstream signaling that mediates positive T cell selection, as well as T cell differentiation and CD4+ Th2/Th17 (Huang, Jeong, Kannan, Huang, & August, 2014). The potential role of bovine ITK in MAP infection has been inferred from its important role in the secretion of IL-2, Th2, IL-4, IL-5, IL-13 cytokines, and interferon-gamma (Mueller & August, 2003).

**Sienna3 module**

In terms of functional analysis, the sienna3 module was associated with different immune pathways such as “positive regulation of tumor necrosis factor production,” “positive regulation of autophagy,” “response to type I interferon,” and “toll-like receptor signaling pathway”. Several hub genes in this module were involved in JD as displayed by other researchers. These hub genes include RPS6KA2 (Brito et al., 2018), ZBTB44 (Malvisi et al., 2016), RORA (Mallikarjunappa et al., 2018), MX1 (P. Gupta et al., 2019), SLC2A3 (Ariel et al., 2019), and VEGFA (David, Barkema, Mortier, et al., 2014). ZBTB44 and RORA were also TF, and SLC2A3 and VEGFA were hub-hub genes. MX1 gene is involved in immune-related functions and organelles fission (P. Gupta et al., 2019). The SLC2A3 gene is one of the genes encoding glucose transporter that is classically associated with activated macrophages (Ariel et al., 2019).

**Grey60 module**

The grey60 module represented the functional enriched terms such as “phagocytosis,” and “toll-like receptor 4 signaling pathway”. The genes that were related to JD as other researchers reported are as follows: USO1 hub gene plays role in vesicle membrane and cytoplasmic vesicle membrane and it was reported to be associated with MAP susceptibility (Kiser et al., 2018). GTF2I TF gene codes for a protein that may be involved in the immune defense against MAP infection or related to response to bacterial infection (Del Corvo et al., 2017). Many important cellular processes are affected by this gene, including apoptosis, ligand-receptor neuroactive interaction, and calcium signaling pathway, ribosomal cytokine-cytokine receptor interaction pathway, cleavage binding, and adhesive binding (Chimge, Makeyev, Ruddle, & Bayarsaihan, 2008). This gene probably regulates a subset of stress-induced genes in the endoplasmic reticulum that are components of a defense mechanism that may be associated with apoptosis in macrophages. GTF2I gene may participate in pathogenesis and resistance of mycobacteria in macrophages associated with MAP infection (Lim et al., 2011).

**Lightcyan1 module**

Functional enrichment analysis indicated that genes in the lightcyan1 module were enriched in positive regulation of signal transduction pathway. Investigation of the association of genes with JD in this module revealed some related genes. DCHS1 hub gene functions in the inflammatory response and showed increased expression in MAP-infected animals (Malvisi et al., 2016). RXRA, TF gene, has a role in leukocyte migration, the concentration of lipid, steroid binding, and had differential expression in infected cows (Alluwaimi & Badi, 2015). IRF3 gene is another TF which is one of the interferon regulatory factors and has an immune regulatory function. This gene was downregulated in infected animals (Park et al., 2018).

**Lightgreen module**

Based on GO results, in the lightgreen module genes were enriched in “regulation of T cell proliferation,” and “leukocyte cell-cell adhesion”. The following hub genes in the lightgreen module were associated with JD according to previous studies. FERMT3 hub-hub gene has a role in cell-to-cell signaling pathway and was upregulated in infected animals (David, Barkema, Mortier, et al., 2014). NFKBIB hub gene participates in the chemokine signaling pathway and NOD-like receptor signaling pathway (P. Gupta et al., 2019). This gene was significantly up-regulated in the early phase of MAP infection (Khare et al., 2012). ERF is a TF gene and a transcription suppressor. It is focused that ERF has tumor suppressor activity (Armstrong & Hart, 1971). For ERF gene, functions in immune response and macrophage biology have been mentioned and it was introduced as a candidate gene for JD diagnosis (Verykokakis, Papadaki, Vorgia, Le Gallic, & Mavrothalassitis, 2007). This gene showed significant differential expression in infected animals (Malvisi et al., 2020). CSK is related to the regulation of actin cytoskeleton, epithelial cell signaling, and integrin-mediated cell adhesion pathways. All the pathways were active in the early phase of infection and suppressed in intermediated and late stages (Khare et al., 2012). AP2A1 contributes to the movement of vesicles through the cellular membrane and endocytosis (Leong, Ng, & Chu, 2011; Mousavi, Malerød, Berg, & Kjeken, 2004; Traub, 2005). Consequently, the upregulation of AP2A1 may be associated with increased intra-cellular traffic because of MAP phagocytosis (Malvisi et al., 2016).

**Navajowhite2 module**

The most significant GO terms in navajowhite2 module were “interleukin-6,” and “interleukin-8 secretion”, which were associated with JD. The study of the relationship between the genes of this module and JD in the literature showed the association of some hub genes with the disease. NOS2 gene is one of the genes involved in the host immune response to mycobacterial infection (Nicholson et al., 1996). NOS2 is a reactive free radical that acts as a bio-transmitter in various processes including neurotransmission and antimicrobial and antitumor activities. This gene encodes a nitric oxide synthetase that is expressed in the liver and can be induced by a combination of certain lipopolysaccharides and cytokines (Colin Mackintosh, 2012). It was reported that NOS2 is highly expressed at 2 and 6 hours post-infection but it is not differentially expressed at 24 hours post-infection (MacHugh et al., 2012). This situation suggests that MAP infection suppresses expression of this gene within 24 hours after infection via the CD40 signaling pathway (Sommer, Pudrith, Colvin, & Coussens, 2009). PDPK1 gene is one of the genes that has been highly expressed in the middle and late stages of JD in the pathways of cell adhesion molecules (CAM) and integrin-mediated cell adhesion (IMCA), in support of the survival mechanism of MAP. This gene is involved in signaling processes and phosphorylates and activates PKB/AKT (Khare et al., 2012).

**Skyblue3 module**

According to functional analysis, genes in the skyblue3 module were enriched in T cell lineage commitment. SIRT1 hub-hub gene and STAT3 TF gene were also reported associated with JD in previous researches (David, Barkema, Mortier, et al., 2014; Hussain, Shah, Zhao, Sreevatsan, & Zhou, 2016; Khare et al., 2012; Sajiki et al., 2018; Shin et al., 2015; Zanella et al., 2011). STAT3 gene is essential for anti-inflammatory responses, and the pathways in which STAT3 is involved are associated with inhibition of phagosome maturation in mycobacterium-infected macrophages and the development of an immunosuppressive position in granulomas (Lang, 2005). The Th1 response plays a vital role in controlling MAP infection and disease progression, but it induces IL-10 infection, which activates STAT3 through IL-10 receptor signaling, which in turn leads to immunosuppression and bacterial resistance (Hussain et al., 2016; D. J. Weiss, Evanson, de Souza, & Abrahamsen, 2005). In fact, IL-10 negatively regulates apoptosis through STAT3 activation (Donnelly, Dickensheets, & Finbloom, 1999). STAT3 plays an important role as a signaling mediator of IL-6 and IL-10 family members and other cytokines (Kuchipudi, 2015).

**Tan module**

Functional enrichment results indicated that tam module genes were enriched in negative regulation of cytokine-mediated signaling pathway. The hub genes in the tan module that found to be associated with JD are as follows: VIM (Marino et al., 2017), DEF6 (Gao et al., 2018), DFFA (Kabara et al., 2010), SPN (Khare et al., 2012), DEFB4A (Colin Mackintosh, 2012; Purdie et al., 2012; Shin et al., 2015). VIM hub-hub gene in performing apoptosis and DFFA in the suppression of apoptosis had function (Kabara et al., 2010; Marino et al., 2017). DEF6 is one of the immune system-related genes and expresses in high levels in B and T cells (S. Gupta et al., 2003). DFFA gene is involved in the suppression of apoptosis (Kabara et al., 2010). SPN gene encodes the main glycoproteins of thymocytes and T lymphocytes and takes part in the physicochemical properties of the T-cell surface, lectin binding, and in some B lymphocytes. It seems that these roles are important for immune function and may contribute to a physiologic ligand-receptor complex involved in T-cell activation (Khare et al., 2012). DEFB4A works in defensive response to bacteria (Purdie et al., 2012) and is related to the induction of beta-defensin (Shin et al., 2015).

**Brown4 module**

Genes from brown4 module showed enrichment for cell-cell signaling pathway. There were some genes in this module that were related to JD based on other researchers result: STAB1 (David, Barkema, Mortier, et al., 2014), TBCD (Kiser et al., 2018), ARSB (Hempel, Bannantine, & Stabel, 2016a; Purdie et al., 2012), and RETN (Berry, Wu, Venturino, & Talaat, 2018; Shin et al., 2015). STAB1 hub gene has a role in cell to cell signaling which is an immune function (David, Barkema, Mortier, et al., 2014). TBCD hub gene takes part in the negative regulation of cell adhesion. This gene regulates small GTPase activity. Small GTPases like RhoA have been linked to the process by which MAP crosses the intestinal barrier. Mutations that modify cell adhesion can change the integrity of intestinal epithelial barrier and prevent the migration of immune cells to infection sites, which causes an increase in the risk of infection to the host (Kiser et al., 2018). For ARSB hub-hub gene catalytic activity (Purdie et al., 2012), and role in lysosomal Pathway were reported (Hempel et al., 2016b). RETN is another hub-hub, which has potential roles in immune (Berry et al., 2018)), inhibition of functions in free radical scavenging, lipid metabolism, and small molecules biochemistry (Shin et al., 2015).

**Thistle2 module**

NRROS and PRXL2A hub genes were enriched in myeloid leukocyte differentiation. In this module, there were hub genes associated with JD according to other researchers’ results: DUSP2 (Hossain et al., 2006), STAT1 (Coussens et al., 2004), SCARB2 (Marino et al., 2017). DUSP2 gene is a phosphatase that is involved in post-infection anti-inflammatory processes (Hossain et al., 2006). STAT1 TF gene has a role in regulating the transcription and signaling of interferon-gamma (Coussens et al., 2004). SCARB2 gene is a lysosomal membrane protein (Marino et al., 2017).

**References**

Alluwaimi, A. M., & Badi, F. A. (2015). Mycobacterium avium subspecies paratuberculosis infection in naturally infected cattle is associated with an upregulation of lipid metabolism gene expression.

Alonso-Hearn, M., Canive, M., Blanco-Vazquez, C., Torremocha, R., Balseiro, A., Amado, J., . . . Casais, R. (2019). RNA-Seq analysis of ileocecal valve and peripheral blood from Holstein cattle infected with Mycobacterium avium subsp. paratuberculosis revealed dysregulation of the CXCL8/IL8 signaling pathway. *Scientific reports, 9*(1), 1-17.

Alpay, F., Zare, Y., Kamalludin, M. H., Huang, X., Shi, X., Shook, G. E., . . . Kirkpatrick, B. W. (2014). Genome-wide association study of susceptibility to infection by Mycobacterium avium subspecies paratuberculosis in Holstein cattle. *PLoS One, 9*(12).

Ariel, O., Gendron, D., Dudemaine, P.-L., Gévry, N., Ibeagha-Awemu, E. M., & Bissonnette, N. (2019). Transcriptome profiling of bovine macrophages infected by Mycobacterium avium ssp. paratuberculosis depicts foam cell and innate immune tolerance phenotypes. *Frontiers in immunology, 10*, 2874.

Armstrong, J., & Hart, P. D. A. (1971). Response of cultured macrophages to Mycobacterium tuberculosis, with observations on fusion of lysosomes with phagosomes. *The Journal of experimental medicine, 134*(3), 713-740.

Arsenault, R. J., Maattanen, P., Daigle, J., Potter, A., Griebel, P., & Napper, S. (2014). From mouth to macrophage: mechanisms of innate immune subversion by Mycobacterium avium subsp. paratuberculosis. *Veterinary research, 45*(1), 54.

Bassey, E., & Collins, M. T. (1997). Study of T-lymphocyte subsets of healthy and Mycobacterium avium subsp. paratuberculosis-infected cattle. *Infection and immunity, 65*(11), 4869-4872.

Berry, A., Wu, C.-w., Venturino, A. J., & Talaat, A. M. (2018). Biomarkers for early stages of Johne’s Disease infection and immunization in goats. *Frontiers in microbiology, 9*, 2284.

Blumerman, S. L., Herzig, C. T., Wang, F., Coussens, P. M., & Baldwin, C. L. (2007). Comparison of gene expression by co-cultured WC1+ γδ and CD4+ αβ T cells exhibiting a recall response to bacterial antigen. *Molecular immunology, 44*(8), 2023-2035.

Brito, L. F., Mallikarjunappa, S., Sargolzaei, M., Koeck, A., Chesnais, J., Schenkel, F. S., . . . Karrow, N. A. (2018). The genetic architecture of milk ELISA scores as an indicator of Johne's disease (paratuberculosis) in dairy cattle. *Journal of dairy science, 101*(11), 10062-10075.

Brown, M. S., & Goldstein, J. L. (1986). A receptor-mediated pathway for cholesterol homeostasis. *Science, 232*(4746), 34-47.

Buchsbaum, S., Bercovich, B., Ziv, T., & Ciechanover, A. (2012). Modification of the inflammatory mediator LRRFIP2 by the ubiquitin-like protein FAT10 inhibits its activity during cellular response to LPS. *Biochemical and biophysical research communications, 428*(1), 11-16.

Cannons, J. L., & Schwartzberg, P. L. (2004). Fine-tuning lymphocyte regulation: what’s new with tyrosine kinases and phosphatases? *Current opinion in immunology, 16*(3), 296-303.

Casey, M. E., Meade, K. G., Nalpas, N. C., Taraktsoglou, M., Browne, J. A., Killick, K. E., . . . Magee, D. A. (2015). Analysis of the bovine monocyte-derived macrophage response to Mycobacterium avium subspecies paratuberculosis infection using RNA-seq. *Frontiers in immunology, 6*, 23.

Chen, G., Shaw, M. H., Kim, Y.-G., & Nuñez, G. (2009). NOD-like receptors: role in innate immunity and inflammatory disease. *Annual Review of Pathology: Mechanisms of Disease, 4*, 365-398.

Chen, Z., Yuan, Y.-C., Wang, Y., Liu, Z., Chan, H. J., & Chen, S. (2015). Down-regulation of programmed cell death 4 (PDCD4) is associated with aromatase inhibitor resistance and a poor prognosis in estrogen receptor-positive breast cancer. *Breast cancer research and treatment, 152*(1), 29-39.

Chimge, N.-O., Makeyev, A. V., Ruddle, F. H., & Bayarsaihan, D. (2008). Identification of the TFII-I family target genes in the vertebrate genome. *Proceedings of the National Academy of Sciences, 105*(26), 9006-9010.

Clark, D., Koziczkowski, J., Radcliff, R., Carlson, R., & Ellingson, J. (2008). Detection of Mycobacterium avium subspecies paratuberculosis: comparing fecal culture versus serum enzyme-linked immunosorbent assay and direct fecal polymerase chain reaction. *Journal of dairy science, 91*(7), 2620-2627.

Coussens, P. M., Jeffers, A., & Colvin, C. (2004). Rapid and transient activation of gene expression in peripheral blood mononuclear cells from Johne's disease positive cows exposed to Mycobacterium paratuberculosis in vitro. *Microbial pathogenesis, 36*(2), 93-108.

Coussens, P. M., Pudrith, C. B., Skovgaard, K., Ren, X., Suchyta, S. P., Stabel, J. R., & Heegaard, P. M. (2005). Johne's disease in cattle is associated with enhanced expression of genes encoding IL-5, GATA-3, tissue inhibitors of matrix metalloproteinases 1 and 2, and factors promoting apoptosis in peripheral blood mononuclear cells. *Veterinary immunology and immunopathology, 105*(3-4), 221-234.

David, J., Barkema, H. W., & De Buck, J. (2014). Gene-expression profiling of calves 6 and 9 months after inoculation with Mycobacterium avium subspecies paratuberculosis. *Veterinary research, 45*(1), 96.

David, J., Barkema, H. W., Mortier, R., Ghosh, S., & De Buck, J. (2014). Gene expression profiling and putative biomarkers of calves 3 months after infection with Mycobacterium avium subspecies paratuberculosis. *Veterinary immunology and immunopathology, 160*(1-2), 107-117.

De Chastellier, C., & Thilo, L. (2006). Cholesterol depletion in Mycobacterium avium‐infected macrophages overcomes the block in phagosome maturation and leads to the reversible sequestration of viable mycobacteria in phagolysosome‐derived autophagic vacuoles. *Cellular microbiology, 8*(2), 242-256.

Del Corvo, M., Luini, M., Stella, A., Pagnacco, G., Ajmone-Marsan, P., Williams, J. L., & Minozzi, G. (2017). Identification of additional loci associated with antibody response to Mycobacterium avium ssp. Paratuberculosis in cattle by GSEA–SNP analysis. *Mammalian Genome, 28*(11-12), 520-527.

Delgado, M. A., Elmaoued, R. A., Davis, A. S., Kyei, G., & Deretic, V. (2008). Toll‐like receptors control autophagy. *The EMBO journal, 27*(7), 1110-1121.

Dobson, B. J. (2012). *Immunological Parameters of Resistance and Susceptibility to Johne’s Disease in Red Deer (Cervus elaphus).* University of Otago.

Donnelly, R. P., Dickensheets, H., & Finbloom, D. S. (1999). The interleukin-10 signal transduction pathway and regulation of gene expression in mononuclear phagocytes. *Journal of interferon & cytokine research, 19*(6), 563-573.

Ewalt, K. L., & Schimmel, P. (2002). Activation of angiogenic signaling pathways by two human tRNA synthetases. *Biochemistry, 41*(45), 13344-13349.

Ferwerda, G., Kullberg, B. J., De Jong, D. J., Girardin, S. E., Langenberg, D. M., Van Crevel, R., . . . Netea, M. G. (2007). Mycobacterium paratuberculosis is recognized by Toll‐like receptors and NOD2. *Journal of leukocyte biology, 82*(4), 1011-1018.

Gao, Y., Jiang, J., Yang, S., Cao, J., Han, B., Wang, Y., . . . Zhang, Q. (2018). Genome-wide association study of Mycobacterium avium subspecies Paratuberculosis infection in Chinese Holstein. *BMC genomics, 19*(1), 1-10.

Gossner, A., Watkins, C., Chianini, F., & Hopkins, J. (2017). Pathways and genes associated with immune dysfunction in sheep paratuberculosis. *Scientific reports, 7*, 46695.

Gu, Y., Chae, H.-D., Siefring, J. E., Jasti, A. C., Hildeman, D. A., & Williams, D. A. (2006). RhoH GTPase recruits and activates Zap70 required for T cell receptor signaling and thymocyte development. *Nature immunology, 7*(11), 1182-1190.

Gupta, P., Peter, S., Jung, M., Lewin, A., Hemmrich-Stanisak, G., Franke, A., . . . Sharbati, S. (2019). Analysis of long non-coding RNA and mRNA expression in bovine macrophages brings up novel aspects of Mycobacterium avium subspecies paratuberculosis infections. *Scientific reports, 9*(1), 1-14.

Gupta, S., Lee, A., Hu, C., Fanzo, J., Goldberg, I., Cattoretti, G., & Pernis, A. B. (2003). Molecular cloning of IBP, a SWAP-70 homologous GEF, which is highly expressed in the immune system. *Human immunology, 64*(4), 389-401.

Hempel, R. J., Bannantine, J. P., & Stabel, J. R. (2016a). Transcriptional profiling of ileocecal valve of Holstein dairy cows infected with Mycobacterium avium subsp. paratuberculosis. *PLoS One, 11*(4).

Hempel, R. J., Bannantine, J. P., & Stabel, J. R. (2016b). Transcriptional Profiling of Ileocecal Valve of Holstein Dairy Cows Infected with Mycobacterium avium subsp. Paratuberculosis. *PLoS One, 11*(4), e0153932.

Henckaerts, L., Pierik, M., Joossens, M., Ferrante, M., Rutgeerts, P., & Vermeire, S. (2007). Mutations in pattern recognition receptor genes modulate seroreactivity to microbial antigens in patients with inflammatory bowel disease. *Gut, 56*(11), 1536-1542.

Honda, K., & Taniguchi, T. (2006). IRFs: master regulators of signalling by Toll-like receptors and cytosolic pattern-recognition receptors. *Nature Reviews Immunology, 6*(9), 644-658.

Hossain, H., Tchatalbachev, S., & Chakraborty, T. (2006). Host gene expression profiling in pathogen–host interactions. *Current opinion in immunology, 18*(4), 422-429.

Huang, W., Jeong, A.-R., Kannan, A. K., Huang, L., & August, A. (2014). IL-2–inducible T cell kinase tunes T regulatory cell development and is required for suppressive function. *The Journal of Immunology, 193*(5), 2267-2272.

Hussain, T., Shah, S. Z. A., Zhao, D., Sreevatsan, S., & Zhou, X. (2016). The role of IL-10 in Mycobacterium avium subsp. paratuberculosis infection. *Cell Communication and Signaling, 14*(1), 29.

Huynh, K. K., Gershenzon, E., & Grinstein, S. (2008). Cholesterol accumulation by macrophages impairs phagosome maturation. *Journal of Biological Chemistry, 283*(51), 35745-35755.

Ibeagha-Awemu, E., Do, D., Dudemaine, P., & Bissonnette, N. (2018). PSXIV-17 Gene co-expression network analysis identifies important modules and genes for cow’s response to Mycobacterium avium ssp. paratuberculosis infection in the small intestine. *Journal of animal science, 96*(suppl_3), 39-39.

Jayachandran, R., & Pieters, J. (2015). Regulation of immune cell homeostasis and function by coronin 1. *International immunopharmacology, 28*(2), 825-828.

Jayachandran, R., Sundaramurthy, V., Combaluzier, B., Mueller, P., Korf, H., Huygen, K., . . . Pieters, J. (2007). Survival of mycobacteria in macrophages is mediated by coronin 1-dependent activation of calcineurin. *Cell, 130*(1), 37-50.

Johansen, M. D., de Silva, K., Plain, K. M., Whittington, R. J., & Purdie, A. C. (2019). Mycobacterium avium subspecies paratuberculosis is able to manipulate host lipid metabolism and accumulate cholesterol within macrophages. *Microbial pathogenesis, 130*, 44-53.

Johnson, L. A., & Jackson, D. G. (2014). Control of dendritic cell trafficking in lymphatics by chemokines. *Angiogenesis, 17*(2), 335-345.

Kabara, E., Kloss, C. C., Wilson, M., Tempelman, R. J., Sreevatsan, S., Janagama, H., & Coussens, P. M. (2010). A large-scale study of differential gene expression in monocyte-derived macrophages infected with several strains of Mycobacterium avium subspecies paratuberculosis. *Briefings in functional genomics, 9*(3), 220-237.

Kawai, T., & Akira, S. (2007). Signaling to NF-κB by Toll-like receptors. *Trends in molecular medicine, 13*(11), 460-469.

Keown, D. A., Collings, D. A., & Keenan, J. I. (2012). Uptake and persistence of Mycobacterium avium subsp. paratuberculosis in human monocytes. *Infection and immunity, 80*(11), 3768-3775.

Khare, S., Drake, K. L., Lawhon, S. D., Nunes, J. E., Figueiredo, J. F., Rossetti, C. A., . . . Adams, L. G. (2016). Systems analysis of early host gene expression provides clues for transient Mycobacterium avium ssp avium vs. persistent Mycobacterium avium ssp paratuberculosis intestinal infections. *PLoS One, 11*(9).

Khare, S., Lawhon, S. D., Drake, K. L., Nunes, J. E., Figueiredo, J. F., Rossetti, C. A., . . . Galindo, C. L. (2012). Systems biology analysis of gene expression during in vivo Mycobacterium avium paratuberculosis enteric colonization reveals role for immune tolerance. *PLoS One, 7*(8).

Kiser, J., Neupane, M., White, S., & Neibergs, H. (2018). Identification of genes associated with susceptibility to Mycobacterium avium ssp. paratuberculosis (Map) tissue infection in Holstein cattle using gene set enrichment analysis–SNP. *Mammalian Genome, 29*(7-8), 539-549.

Kiser, J., White, S., Johnson, K., Hoff, J., Taylor, J., & Neibergs, H. (2017). Identification of loci associated with susceptibility to Mycobacterium avium subspecies paratuberculosis (Map) tissue infection in cattle. *Journal of animal science, 95*(3), 1080-1091.

Kuchipudi, S. V. (2015). The complex role of STAT3 in viral infections. *Journal of immunology research, 2015*.

Lande, R., Giacomini, E., Grassi, T., Remoli, M. E., Iona, E., Miettinen, M., . . . Coccia, E. M. (2003). IFN-αβ released by Mycobacterium tuberculosis-infected human dendritic cells induces the expression of CXCL10: selective recruitment of NK and activated T cells. *The Journal of Immunology, 170*(3), 1174-1182.

Lang, R. (2005). Tuning of macrophage responses by Stat3-inducing cytokines: molecular mechanisms and consequences in infection. *Immunobiology, 210*(2-4), 63-76.

Leite, F. L., Eslabão, L. B., Pesch, B., Bannantine, J. P., Reinhardt, T. A., & Stabel, J. R. (2015). ZAP-70, CTLA-4 and proximal T cell receptor signaling in cows infected with Mycobacterium avium subsp. paratuberculosis. *Veterinary immunology and immunopathology, 167*(1-2), 15-21.

Leong, K. L. J., Ng, M. M.-L., & Chu, J. J. H. (2011). The essential role of clathrin-mediated endocytosis in the infectious entry of human enterovirus 71. *Journal of Biological Chemistry, 286*(1), 309-321.

Li, J. Z. H., Gao, W., Ho, W.-K., Lei, W. B., Wei, W. I., Chan, J. Y.-W., & Wong, T.-S. (2016). The clinical association of programmed cell death protein 4 (PDCD4) with solid tumors and its prognostic significance: a meta-analysis. *Chinese journal of cancer, 35*(1), 95.

Liang, X., Xu, Z., Yuan, M., Zhang, Y., Zhao, B., Wang, J., . . . Li, G. (2016). MicroRNA-16 suppresses the activation of inflammatory macrophages in atherosclerosis by targeting PDCD4. *International journal of molecular medicine, 37*(4), 967-975.

Lim, Y.-J., Choi, J.-A., Choi, H.-H., Cho, S.-N., Kim, H.-J., Jo, E.-K., . . . Song, C.-H. (2011). Endoplasmic reticulum stress pathway-mediated apoptosis in macrophages contributes to the survival of Mycobacterium tuberculosis. *PLoS One, 6*(12).

Liu, X., Zhang, P., Bao, Y., Han, Y., Wang, Y., Zhang, Q., . . . Li, N. (2013). Zinc finger protein ZBTB20 promotes toll-like receptor-triggered innate immune responses by repressing IκBα gene transcription. *Proceedings of the National Academy of Sciences, 110*(27), 11097-11102.

Lou, Y., & Liu, S. (2011). The TIPE (TNFAIP8) family in inflammation, immunity, and cancer. *Molecular immunology, 49*(1-2), 4-7.

Luzio, J. P., Pryor, P. R., & Bright, N. A. (2007). Lysosomes: fusion and function. *Nature reviews Molecular cell biology, 8*(8), 622-632.

MacHugh, D. E., Taraktsoglou, M., Killick, K. E., Nalpas, N. C., Browne, J. A., Park, S. D., . . . Magee, D. A. (2012). Pan-genomic analysis of bovine monocyte-derived macrophage gene expression in response to in vitro infection with Mycobacterium avium subspecies paratuberculosis. *Veterinary research, 43*(1), 25.

Mackintosh, C., Griffin, J., Scott, I., O’Brien, R., Stanton, J., MacLean, P., & Brauning, R. (2016). SOLiD SAGE sequencing shows differential gene expression in jejunal lymph node samples of resistant and susceptible red deer (Cervus elaphus) challenged with Mycobacterium avium subsp. paratuberculosis. *Veterinary immunology and immunopathology, 169*, 102-110.

Mackintosh, C. (2012), A. Review of Genetics of host resistance/susceptibility to paratuberculosis. Invermay Agricultural Centre, Mosgiel, New Zealand, jdrc.co.nz.

Mallikarjunappa, S., Sargolzaei, M., Brito, L. F., Meade, K. G., Karrow, N., & Pant, S. (2018). Uncovering quantitative trait loci associated with resistance to Mycobacterium avium ssp. paratuberculosis infection in Holstein cattle using a high-density single nucleotide polymorphism panel. *Journal of dairy science, 101*(8), 7280-7286.

Malvisi, M., Curti, N., Remondini, D., De Iorio, M. G., Palazzo, F., Gandini, G., . . . Minozzi, G. (2020). Combinatorial Discriminant Analysis Applied to RNAseq Data Reveals a Set of 10 Transcripts as Signatures of Exposure of Cattle to Mycobacterium avium subsp. paratuberculosis. *Animals, 10*(2), 253.

Malvisi, M., Palazzo, F., Morandi, N., Lazzari, B., Williams, J. L., Pagnacco, G., & Minozzi, G. (2016). Responses of bovine innate immunity to Mycobacterium avium subsp. paratuberculosis infection revealed by changes in gene expression and levels of microRNA. *PLoS One, 11*(10).

Marino, R., Capoferri, R., Panelli, S., Minozzi, G., Strozzi, F., Trevisi, E., . . . Williams, J. L. (2017). Johne’s disease in cattle: an in vitro model to study early response to infection of Mycobacterium avium subsp. paratuberculosis using RNA-seq. *Molecular immunology, 91*, 259-271.

McGarvey, J., Wagner, D., & Bermudez, L. (2004). Differential gene expression in mononuclear phagocytes infected with pathogenic and non‐pathogenic mycobacteria. *Clinical & Experimental Immunology, 136*(3), 490-500.

McGovern, S., Purfield, D., Ring, S., Carthy, T., Graham, D., & Berry, D. P. (2019). Candidate genes associated with the heritable humoral response to Mycobacterium avium ssp. paratuberculosis in dairy cows have factors in common with gastrointestinal diseases in humans. *Journal of dairy science, 102*(5), 4249-4263.

McNab, F., Mayer-Barber, K., Sher, A., Wack, A., & O'garra, A. (2015). Type I interferons in infectious disease. *Nature Reviews Immunology, 15*(2), 87-103.

McNees, A. L., Markesich, D., Zayyani, N. R., & Graham, D. Y. (2015). Mycobacterium paratuberculosis as a cause of Crohn’s disease. *Expert review of gastroenterology & hepatology, 9*(12), 1523-1534.

McSpadden, K., Caires, K., & Zanella, R. (2013). The effect of Mycobacterium avium subspecies paratuberculosis exposure on animal health. *Acta Scientiae Veterinariae, 41*(1), 1-10.

Mellins, E. D., & Stern, L. J. (2014). HLA-DM and HLA-DO, key regulators of MHC-II processing and presentation. *Current opinion in immunology, 26*, 115-122.

Meyers, J. H., Chakravarti, S., Schlesinger, D., Illes, Z., Waldner, H., Umetsu, S. E., . . . DeKruyff, R. H. (2005). TIM-4 is the ligand for TIM-1, and the TIM-1–TIM-4 interaction regulates T cell proliferation. *Nature immunology, 6*(5), 455-464.

Moreland, J. G., Davis, A. P., Bailey, G., Nauseef, W. M., & Lamb, F. S. (2006). Anion channels, including ClC-3, are required for normal neutrophil oxidative function, phagocytosis, and transendothelial migration. *Journal of Biological Chemistry, 281*(18), 12277-12288.

Mousavi, S. A., Malerød, L., Berg, T., & Kjeken, R. (2004). Clathrin-dependent endocytosis. *Biochemical Journal, 377*(Pt 1), 1.

Mueller, C., & August, A. (2003). Attenuation of immunological symptoms of allergic asthma in mice lacking the tyrosine kinase ITK. *The Journal of Immunology, 170*(10), 5056-5063.

Neibergs, H. L., Settles, M. L., Whitlock, R. H., & Taylor, J. F. (2010). GSEA-SNP identifies genes associated with Johne’s disease in cattle. *Mammalian Genome, 21*(7-8), 419-425.

Nicholson, S., Bonecini-Almeida, M. d. G., Lapa e Silva, J., Nathan, C., Xie, Q., Mumford, R., . . . Boechat, N. (1996). Inducible nitric oxide synthase in pulmonary alveolar macrophages from patients with tuberculosis. *The Journal of experimental medicine, 183*(5), 2293-2302.

O'Leary, N. A., Wright, M. W., Brister, J. R., Ciufo, S., Haddad, D., McVeigh, R., . . . Ako-Adjei, D. (2016). Reference sequence (RefSeq) database at NCBI: current status, taxonomic expansion, and functional annotation. *Nucleic acids research, 44*(D1), D733-D745.

Paixão, T. A., Poester, F. P., Neta, A. V. C., Borges, Á. M., Lage, A. P., & Santos, R. L. (2007). NRAMP1 3′ untranslated region polymorphisms are not associated with natural resistance to Brucella abortus in cattle. *Infection and immunity, 75*(5), 2493-2499.

Pant, S. D., Schenkel, F. S., Verschoor, C. P., You, Q., Kelton, D. F., Moore, S. S., & Karrow, N. A. (2010). A principal component regression based genome wide analysis approach reveals the presence of a novel QTL on BTA7 for MAP resistance in holstein cattle. *Genomics, 95*(3), 176-182.

Park, H.-E., Park, H.-T., Jung, Y. H., & Yoo, H. S. (2018). Gene expression profiles of immune-regulatory genes in whole blood of cattle with a subclinical infection of Mycobacterium avium subsp. paratuberculosis. *PLoS One, 13*(4).

Periasamy, S., Tripathi, B. N., & Singh, N. (2013). Mechanisms of Mycobacterium avium subsp. paratuberculosis induced apoptosis and necrosis in bovine macrophages. *Veterinary microbiology, 165*(3-4), 392-401.

Pinedo, P. J., Buergelt, C. D., Donovan, G. A., Melendez, P., Morel, L., Wu, R., . . . Rae, D. O. (2009). Candidate gene polymorphisms (BoIFNG, TLR4, SLC11A1) as risk factors for paratuberculosis infection in cattle. *Preventive veterinary medicine, 91*(2-4), 189-196.

Podinovskaia, M., Lee, W., Caldwell, S., & Russell, D. G. (2013). Infection of macrophages with M ycobacterium tuberculosis induces global modifications to phagosomal function. *Cellular microbiology, 15*(6), 843-859.

Pos, W., Sethi, D. K., & Wucherpfennig, K. W. (2013). Mechanisms of peptide repertoire selection by HLA-DM. *Trends in immunology, 34*(10), 495-501.

Purdie, A. C., Plain, K. M., Begg, D. J., de Silva, K., & Whittington, R. J. (2012). Expression of genes associated with the antigen presentation and processing pathway are consistently regulated in early Mycobacterium avium subsp. paratuberculosis infection. *Comparative immunology, microbiology and infectious diseases, 35*(2), 151-162.

Quesniaux, V., Fremond, C., Jacobs, M., Parida, S., Nicolle, D., Yeremeev, V., . . . Drennan, M. (2004). Toll-like receptor pathways in the immune responses to mycobacteria. *Microbes and infection, 6*(10), 946-959.

Rathnaiah, G., Zinniel, D. K., Bannantine, J. P., Stabel, J. R., Gröhn, Y. T., Collins, M. T., & Barletta, R. G. (2017). Pathogenesis, molecular genetics, and genomics of Mycobacterium avium subsp. paratuberculosis, the etiologic agent of Johne’s disease. *Frontiers in veterinary science, 4*, 187.

Reddacliff, L., Beh, K., McGregor, H., & Whittington, R. (2005). A preliminary study of possible genetic influences on the susceptibility of sheep to Johne's disease. *Australian veterinary journal, 83*(7), 435-441.

Reiling, N., Blumenthal, A., Flad, H.-D., Ernst, M., & Ehlers, S. (2001). Mycobacteria-induced TNF-α and IL-10 formation by human macrophages is differentially regulated at the level of mitogen-activated protein kinase activity. *The Journal of Immunology, 167*(6), 3339-3345.

Robbins, P. D., & Morelli, A. E. (2014). Regulation of immune responses by extracellular vesicles. *Nature Reviews Immunology, 14*(3), 195-208.

Roussey, J. A., Oliveira, L. J., Langohr, I. M., Sledge, D. G., & Coussens, P. M. (2016). Regulatory T cells and immune profiling in Johne’s disease lesions. *Veterinary immunology and immunopathology, 181*, 39-50.

Ruiz-Larrañaga, O., Garrido, J., Manzano, C., Iriondo, M., Molina, E., Gil, A., . . . Estonba, A. (2010). Identification of single nucleotide polymorphisms in the bovine solute carrier family 11 member 1 (SLC11A1) gene and their association with infection by Mycobacterium avium subspecies paratuberculosis. *Journal of dairy science, 93*(4), 1713-1721.

Rybakin, V., & Clemen, C. S. (2005). Coronin proteins as multifunctional regulators of the cytoskeleton and membrane trafficking. *Bioessays, 27*(6), 625-632.

Sahu, N., & August, A. (2009). ITK inhibitors in inflammation and immune-mediated disorders. *Current topics in medicinal chemistry, 9*(8), 690-703.

Sahu, N., Mueller, C., Fischer, A., & August, A. (2008). Differential sensitivity to Itk kinase signals for T helper 2 cytokine production and chemokine-mediated migration. *The Journal of Immunology, 180*(6), 3833-3838.

Sajiki, Y., Konnai, S., Okagawa, T., Nishimori, A., Maekawa, N., Goto, S., . . . Kagawa, Y. (2018). Prostaglandin E2 induction suppresses the Th1 immune responses in cattle with Johne's disease. *Infection and immunity, 86*(5), e00910-00917.

Sallam, A. M., Zare, Y., Shook, G., Collins, M., & Kirkpatrick, B. W. (2018). A positional candidate gene association analysis of susceptibility to paratuberculosis on bovine chromosome 7. *Infection, Genetics and Evolution, 65*, 163-169.

Schaeuble, K., Hauser, M. A., Singer, E., Groettrup, M., & Legler, D. F. (2011). Cross-talk between TCR and CCR7 signaling sets a temporal threshold for enhanced T lymphocyte migration. *The Journal of Immunology, 187*(11), 5645-5652.

Settles, M., Zanella, R., McKay, S., Schnabel, R., Taylor, J., Whitlock, R., . . . Neibergs, H. (2009). A whole genome association analysis identifies loci associated with Mycobacterium avium subsp. paratuberculosis infection status in US holstein cattle. *Animal genetics, 40*(5), 655-662.

Seya, T., Oshiumi, H., Sasai, M., Akazawa, T., & Matsumoto, M. (2005). TICAM-1 and TICAM-2: toll-like receptor adapters that participate in induction of type 1 interferons. *The international journal of biochemistry & cell biology, 37*(3), 524-529.

Sheedy, F. J., Palsson-McDermott, E., Hennessy, E. J., Martin, C., O'leary, J. J., Ruan, Q., . . . O'neill, L. A. (2010). Negative regulation of TLR4 via targeting of the proinflammatory tumor suppressor PDCD4 by the microRNA miR-21. *Nature immunology, 11*(2), 141.

Shin, M.-K., Park, H.-T., Shin, S. W., Jung, M., Im, Y. B., Park, H.-E., . . . Yoo, H. S. (2015). Whole-blood gene-expression profiles of cows infected with Mycobacterium avium subsp. paratuberculosis reveal changes in immune response and lipid metabolism. *J Microbiol Biotechnol, 25*, 255-267.

Smeed, J., Watkins, C., Gossner, A., & Hopkins, J. (2010). Expression profiling reveals differences in immuno-inflammatory gene expression between the two disease forms of sheep paratuberculosis. *Veterinary immunology and immunopathology, 135*(3-4), 218-225.

Soe-Lin, S., Sheftel, A. D., Wasyluk, B., & Ponka, P. (2008). Nramp1 equips macrophages for efficient iron recycling. *Experimental hematology, 36*(8), 929-937.

Sommer, S., Pudrith, C. B., Colvin, C. J., & Coussens, P. M. (2009). Mycobacterium avium subspecies paratuberculosis suppresses expression of IL-12p40 and iNOS genes induced by signalling through CD40 in bovine monocyte-derived macrophages. *Veterinary immunology and immunopathology, 128*(1-3), 44-52.

Souza, C. D., Evanson, O. A., & Weiss, D. J. (2007). Role of the mitogen-activated protein kinase pathway in the differential response of bovine monocytes to Mycobacterium avium subsp. paratuberculosis and Mycobacterium avium subsp. avium. *Microbes and infection, 9*(14-15), 1545-1552.

Stabel, J. (2000). Transitions in immune responses to Mycobacterium paratuberculosis. *Veterinary microbiology, 77*(3-4), 465-473.

Ström, J. O., Strid, T., & Hammarström, S. (2012). Disruption of the alox5ap gene ameliorates focal ischemic stroke: possible consequence of impaired leukotriene biosynthesis. *BMC neuroscience, 13*(1), 146.

Suzuki, T., Franchi, L., Toma, C., Ashida, H., Ogawa, M., Yoshikawa, Y., . . . Nuñez, G. (2007). Differential regulation of caspase-1 activation, pyroptosis, and autophagy via Ipaf and ASC in Shigella-infected macrophages. *PLoS pathogens, 3*(8).

Thirunavukkarasu, S., Plain, K. M., de Silva, K., Begg, D., Whittington, R. J., & Purdie, A. C. (2014). Expression of genes associated with cholesterol and lipid metabolism identified as a novel pathway in the early pathogenesis of Mycobacterium avium subspecies paratuberculosis-infection in cattle. *Veterinary immunology and immunopathology, 160*(3-4), 147-157.

Tikhanovich, I., Zhao, J., Bridges, B., Kumer, S., Roberts, B., & Weinman, S. A. (2017). Arginine methylation regulates c-Myc–dependent transcription by altering promoter recruitment of the acetyltransferase p300. *Journal of Biological Chemistry, 292*(32), 13333-13344.

Traub, L. M. (2005). Common principles in clathrin-mediated sorting at the Golgi and the plasma membrane. *Biochimica et Biophysica Acta (BBA)-Molecular Cell Research, 1744*(3), 415-437.

Van Hulzen, K., Schopen, G., van Arendonk, J., Nielen, M., Koets, A., Schrooten, C., & Heuven, H. (2012). Genome-wide association study to identify chromosomal regions associated with antibody response to Mycobacterium avium subspecies paratuberculosis in milk of Dutch Holstein-Friesians. *Journal of dairy science, 95*(5), 2740-2748.

Verykokakis, M., Papadaki, C., Vorgia, E., Le Gallic, L., & Mavrothalassitis, G. (2007). The RAS-dependent ERF control of cell proliferation and differentiation is mediated by c-Myc repression. *Journal of Biological Chemistry, 282*(41), 30285-30294.

Wang, T., Lafuse, W. P., Takeda, K., Akira, S., & Zwilling, B. S. (2002). Rapid chromatin remodeling of Toll-like receptor 2 promoter during infection of macrophages with Mycobacterium avium. *The Journal of Immunology, 169*(2), 795-801.

Wang, T., Li, B., Nelson, C. E., & Nabavi, S. (2019). Comparative analysis of differential gene expression analysis tools for single-cell RNA sequencing data. *BMC bioinformatics, 20*(1), 40.

Weil, R., & Israël, A. (2004). T-cell-receptor-and B-cell-receptor-mediated activation of NF-κB in lymphocytes. *Current opinion in immunology, 16*(3), 374-381.

Weiss, D., Evanson, O., & Souza, C. (2006). Mucosal immune response in cattle with subclinical Johne's disease. *Veterinary pathology, 43*(2), 127-135.

Weiss, D., & Souza, C. (2008). Modulation of Mononuclear Phagocyte Function by Mycobacterium avium subsp. paratuberculosis. *Veterinary pathology, 45*(6), 829-841.

Weiss, D. J., Evanson, O. A., de Souza, C., & Abrahamsen, M. S. (2005). A critical role of interleukin-10 in the response of bovine macrophages to infection by Mycobacterium avium subsp paratuberculosis. *American journal of veterinary research, 66*(4), 721-726.

Weiss, D. J., Souza, C. D., Evanson, O. A., Sanders, M., & Rutherford, M. (2008). Bovine monocyte TLR2 receptors differentially regulate the intracellular fate of Mycobacterium avium subsp. paratuberculosis and Mycobacterium avium subsp. avium. *Journal of leukocyte biology, 83*(1), 48-55.

Woo, S.-R., Heintz, J. A., Albrecht, R., Barletta, R. G., & Czuprynski, C. J. (2007). Life and death in bovine monocytes: the fate of Mycobacterium avium subsp. paratuberculosis. *Microbial pathogenesis, 43*(2-3), 106-113.

Wu, C.-w., Livesey, M., Schmoller, S. K., Manning, E. J., Steinberg, H., Davis, W. C., . . . Talaat, A. M. (2007). Invasion and persistence of Mycobacterium avium subsp. paratuberculosis during early stages of Johne's disease in calves. *Infection and immunity, 75*(5), 2110-2119.

Yadav, M., & Schorey, J. S. (2006). The β-glucan receptor dectin-1 functions together with TLR2 to mediate macrophage activation by mycobacteria. *Blood, 108*(9), 3168-3175.

Yagi, R., Zhu, J., & Paul, W. E. (2011). An updated view on transcription factor GATA3-mediated regulation of Th1 and Th2 cell differentiation. *International immunology, 23*(7), 415-420.

Yoo, J.-Y., & Desiderio, S. (2003). Innate and acquired immunity intersect in a global view of the acute-phase response. *Proceedings of the National Academy of Sciences, 100*(3), 1157-1162.

Zaki, M. H., Lamkanfi, M., & Kanneganti, T.-D. (2011). The Nlrp3 inflammasome: contributions to intestinal homeostasis. *Trends in immunology, 32*(4), 171-179.

Zanella, R., Settles, M., McKay, S., Schnabel, R., Taylor, J., Whitlock, R., . . . Neibergs, H. (2011). Identification of loci associated with tolerance to Johne’s disease in Holstein cattle. *Animal genetics, 42*(1), 28-38.

Zare, Y., Shook, G. E., Collins, M. T., & Kirkpatrick, B. W. (2014). Genome-wide association analysis and genomic prediction of Mycobacterium avium subspecies paratuberculosis infection in US Jersey cattle. *PLoS One, 9*(2).

Zhang, R., Li, R., Liu, Y., Li, L., & Tang, Y. (2019). The glycolytic enzyme PFKFB3 controls TNF-α-induced endothelial proinflammatory responses. *Inflammation, 42*(1), 146-155.

Zhang, Y., & Dong, C. (2007). Regulatory mechanisms of mitogen-activated kinase signaling. *Cellular and molecular life sciences, 64*(21), 2771-2789.

Zhang, Y. L., & Dong, C. (2005). MAP kinases in immune responses. *Cell Mol Immunol, 2*(1), 20-27.

Zhao, C., Denison, C., Huibregtse, J. M., Gygi, S., & Krug, R. M. (2005). Human ISG15 conjugation targets both IFN-induced and constitutively expressed proteins functioning in diverse cellular pathways. *Proceedings of the National Academy of Sciences, 102*(29), 10200-10205.
