# Supplementary material for: Integrated Analysis of lncRNAs, mRNAs, and TFs to Identify Regulatory Networks Underlying MAP Infection in Cattle
Source: Front Genet. 2021 Jul 5;12:668448. doi: 10.3389/fgene.2021.668448 (PMC8287970; doi:10.3389/fgene.2021.668448)

**Supplementary File S9:** Enrichment analysis in the non-preserved modules. Visualization of functional enrichment analysis in clusters in the non-preserved modules.


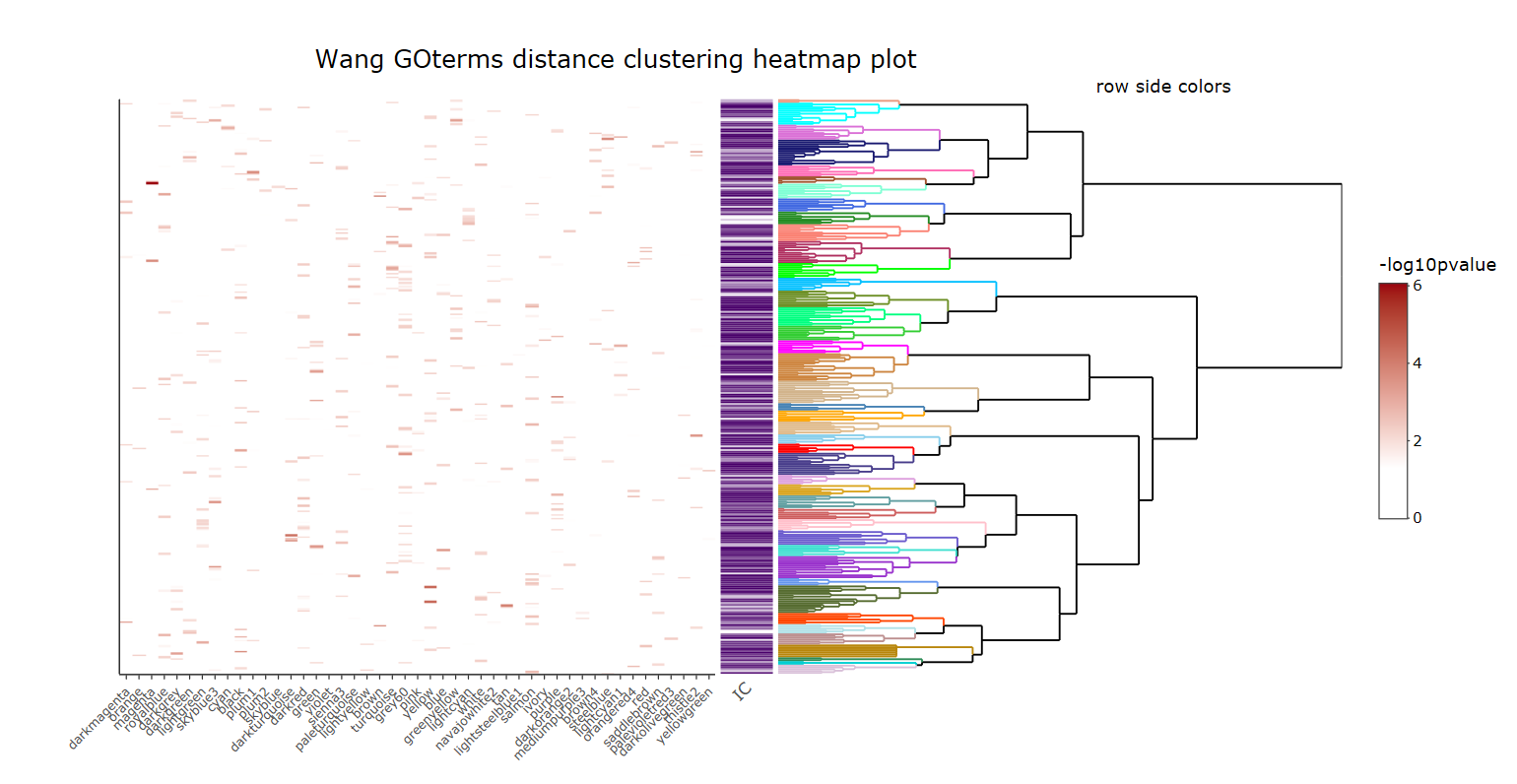

Supplement: Supplementary File 1 — Soft threshold selection process used to obtain the sale free topology index. [file Data_Sheet_1.zip › Data Sheet 1/Supplementary_Materials/Supplementary_File_S9.docx]
